# Comparative metagenomic and metatranscriptomic analyses of microbial communities in acid mine drainage

**DOI:** 10.1038/ismej.2014.245

**Published:** 2014-12-23

**Authors:** Lin-xing Chen, Min Hu, Li-nan Huang, Zheng-shuang Hua, Jia-liang Kuang, Sheng-jin Li, Wen-sheng Shu

**Affiliations:** 1 State Key Laboratory of Biocontrol, Key Laboratory of Biodiversity Dynamics and Conservation of Guangdong Higher Education Institutes, College of Ecology and Evolution, Sun Yat-sen University, Guangzhou, People's Republic of China

## Abstract

The microbial communities in acid mine drainage have been extensively studied to reveal their roles in acid generation and adaption to this environment. Lacking, however, are integrated community- and organism-wide comparative gene transcriptional analyses that could reveal the response and adaptation mechanisms of these extraordinary microorganisms to different environmental conditions. In this study, comparative metagenomics and metatranscriptomics were performed on microbial assemblages collected from four geochemically distinct acid mine drainage (AMD) sites. Taxonomic analysis uncovered unexpectedly high microbial biodiversity of these extremely acidophilic communities, and the abundant taxa of *Acidithiobacillus*, *Leptospirillum* and *Acidiphilium* exhibited high transcriptional activities. Community-wide comparative analyses clearly showed that the AMD microorganisms adapted to the different environmental conditions via regulating the expression of genes involved in multiple *in situ* functional activities, including low-pH adaptation, carbon, nitrogen and phosphate assimilation, energy generation, environmental stress resistance, and other functions. Organism-wide comparative analyses of the active taxa revealed environment-dependent gene transcriptional profiles, especially the distinct strategies used by *Acidithiobacillus ferrivorans* and *Leptospirillum ferrodiazotrophum* in nutrients assimilation and energy generation for survival under different conditions. Overall, these findings demonstrate that the gene transcriptional profiles of AMD microorganisms are closely related to the site physiochemical characteristics, providing clues into the microbial response and adaptation mechanisms in the oligotrophic, extremely acidic environments.

## Introduction

The Earth's extreme environments harbor a wide array of microorganisms with the ability to survive and thrive in physical extremes (Rothschild and Mancinelli, 2001). Analyzing the physiological behaviors and dynamics of these extraordinary communities will reveal the mechanisms that microbes use to respond to environmental change and adapt to the harsh conditions. AMD is generated from the microbially mediated oxidative dissolution of sulfide minerals (Nordstrom and Alpers, 1999) and characterized by low pH and high concentrations of sulfate and metals, representing an extreme environment for life. Two decades of 16S rRNA gene-based molecular surveys have revealed the microbial diversity and ecology of AMD environments (Goebel and Stackebrandt, 1994; Baker and Banfield, 2003; Johnson and Hallberg, 2003; Amaral-Zettler *et al.*, 2011; Kuang *et al.*, 2012). More recently, metagenomic analyses have successfully reconstructed complete or near-complete genomes of dominant AMD microbes directly from environmental samples, possible scenarios for carbon and nitrogen fixation and energy generation, and their potential roles in acid generation have been suggested (Tyson *et al.*, 2004; Baker *et al.*, 2006). On the basis of metagenome-derived genomic information, subsequent metaproteomics have revealed the occurrence of proteins associated with the generation of AMD and the adaptation to extremely acidic habitats (Ram *et al.*, 2005; Baker *et al.*, 2010; Bertin *et al.*, 2011; Méndez-García *et al.*, 2014). Moreover, combined metagenomic and metaproteomic analyses have been conducted to reveal the speciation and evolution in AMD biofilms (Denef and Banfield, 2012). These efforts have largely expanded our knowledge of the microbial ecology, evolution and adaptation in the extreme AMD environment (Denef *et al.*, 2010).

Despite these important advances, the study of gene expression patterns of microbial populations in AMD systems has been limited. Such analyses would provide useful insights into the *in situ* metabolic activities and functional partitioning of microbes in these low-diversity communities. To date, the environmental transcriptomes of microbial assemblages in the Tinto River (Spain) ecosystem have been profiled using *Leptospirillum ferrooxidans* DNA microarrays (Parro and Moreno-Paz, 2003), demonstrating the gene expression profiles of this iron oxidizer to adapt its physiology to changes in physicochemical parameters (Parro *et al.*, 2007) and modes of life (biofilm vs planktonic growth; Moreno-Paz *et al.*, 2010). Another report has attempted integrated community genomics, proteomics and transcriptomics to biofilms in the Richmond Mine AMD system, but, similarly, a single taxon namely the uncultivated new species ‘*Leptospirillum* group IV UBA BS' was targeted in the analysis (Goltsman *et al.*, 2013). Consequently, the *in situ* functional activities of compositionally distinct acidophilic communities and their response to the varying environmental conditions remain unexplored.

We report here the application of combined metagenomic and metatranscriptomic approaches to four AMD microbial assemblages from three geochemically distinct mining sites (Table 1 and Supplementary Figure 1a). By simultaneously investigating the taxonomic composition, gene contents and gene expression patterns, we aimed to reveal the overall functional activities of these extremely acidophilic communities and the specific gene transcriptional behaviors of individual organisms associated with response and adaptation to different environmental conditions.

## Materials and methods

### Sampling and physicochemical characteristics determination

Four AMD samples were collected from three different mine sites in Guangdong Province, China. One of the AMD samples was collected from the mining effluent of the Dabaoshan (DBS) polymetallic ore (24°31'37”N, 113°42'49”E) and another from the acidic drainage pond associated with the tailings impoundment at the Fankou (FK) Pb/Zn mine (25°04'39”N, 113°38'16”E). The other two samples were collected from the Yunfu pyrite mine (22°56'10”N, 112°02'42”E), one from the AMD Yunfu stream (YFS) draining across the surface mining area and the other from the AMD collection Yunfu pond (YFP) downstream before treatment. For DNA/RNA extraction, triplicate samples were collected on site by prefiltering through a 1.6-μm GF/A filter to remove impurities and then filtering through a 0.22-μm polyethersulfone filter using a peristaltic pump. Physicochemical characteristics were determined either on site or in the laboratory as previously described (Kuang *et al.*, 2012). Detailed protocols are provided in Supplementary methods.

### Metagenomic and metatranscriptomic sequencing and bioinformatics analyses

**Figure 1:**
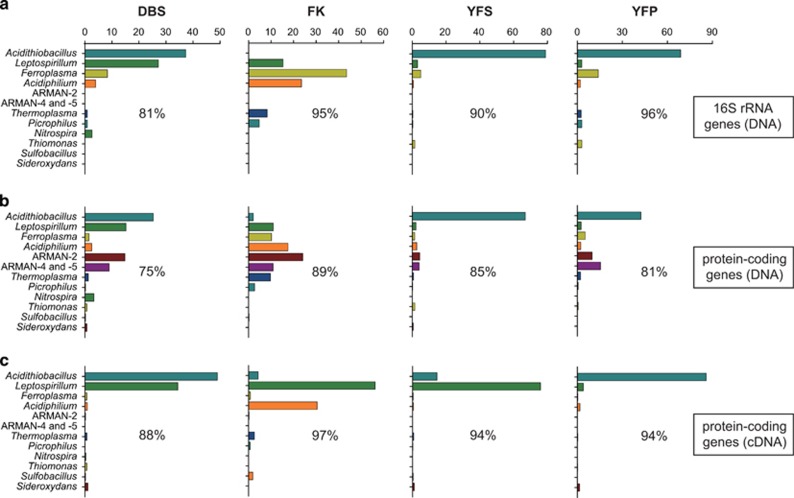
Taxonomic composition and transcriptionally active taxa of the four AMD communities. Community composition analysis was based on taxonomic assignment of (**a**) 16S rRNA gene sequences and (**b**) protein-coding gene sequences in the metagenomic databases, whereas transcriptionally active taxa analysis was based on (**c**) taxonomic assignment of protein-coding gene sequences in the metatranscriptomic databases. Only those genera with a relative abundance of ⩾1% are shown.

For the four AMD samples, DNA and RNA extraction, rRNA subtraction, RNA amplification and cDNA synthesis were performed, and the protocols are detailed in the Supplementary methods. Subsequently, the four metatranscriptomic (cDNA) libraries representing the AMD samples were individually barcoded, and those of DBS and FK, YFS and YFP were, respectively, combined. For each of the two combined cDNA libraries and each of the four metagenomic (DNA) libraries, a whole 454 Titanium run (six runs in total) was sequenced. The pyrosequencing generated a total of 3.5 million DNA and 1.3 million cDNA sequences (Supplementary Table 1). Each of the two combined cDNA libraries (DBS+FK, YFS+YFP) obtained fewer sequences than that of a DNA library (∼72%, Supplementary Table 1), likely because of the incomplete removal of poly(A) tags added for RNA amplification (Shi *et al.*, 2011). By comparing these sequences against a combined 5S, 16S, 18S, 23S and 25S rRNA database from the ARB LSU and SSU databases, 0.2–0.5% of the DNA sequences and 46.7–69.1% of the cDNA sequences were identified as rRNA gene-bearing ones (Supplementary Table 1). The rRNA gene-bearing sequences were compared against the Ribosomal Database Project database (release 10; Cole *et al.*, 2009) to identify 16S rRNA gene-bearing ones, which were further assigned to phylogenetic groups using the Ribosomal Database Project Classifier (Wang *et al.*, 2007; Figure 1a and Supplementary Figure 2). As reported in other metatranscriptomic studies (Stewarts *et al.*, 2010), the depletion of rRNA sequences from the total RNA sample was not sufficient, this was likely due to the unsuccessful removal of archaeal rRNA sequences (Supplementary Figure 2). In each DNA and cDNA non-rRNA data set, replicate sequences sharing 100% nucleotide similarity and length were identified and removed with CD-Hit (Li and Godzik, 2006). The resulting non-rRNA, non-replicate DNA and cDNA sequences were searched for genes in the NCBI non-redundant database, and the results were parsed using the lowest common ancestor algorithm in MEGAN (Huson *et al.*, 2007) to obtain their taxonomic information (Urich *et al.*, 2008; Chen *et al.*, 2013). This obtained ∼500 000 DNA and 50 000 cDNA sequences that matched archaeal and bacterial protein-coding genes for each community, which were retained for further analyses. For functional annotation, the protein-coding sequences were compared against the extended Clusters of Orthologous Groups of proteins (COGs) database (STRING; Franceschini *et al.*, 2013) and the Kyoto Encyclopedia of Genes and Genomes (KEGG) database, and 64.0–83.4% and 75.0–94.1% of which were, respectively, identified as COG and KEGG genes (Supplementary Table 1). See Supplementary methods for detailed protocols. The DNA and cDNA nucleotide sequences are deposited at MG-RAST under the accession numbers of 4568577.3, 4568579.3, 4568579.3, 4568581.3, 4568582.3, 4568583.3, 4568585.3 and 4568661.3.

### Statistical analyses

The relative abundance of a given taxon in a community was calculated as:


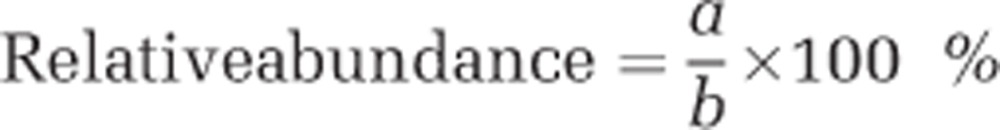


where *a* is the number of sequences assigned to the taxon and *b* is the total number of sequences assigned to all the taxa in the community. Similar calculations were performed for relative abundance of a given gene, COG, COG category, KEGG pathway and KEGG subcategory.

Statistical enrichment of a given gene or COG between two data sets was determined by pairwise comparisons using two-tailed Fisher's exact test, with confidence intervals at 99% significance and Benjamini–Hochberg correction (*P*<0.05).

Principal component analysis was performed using the R package of ‘vegan' (version, 2.0–10). Hierarchical clustering analysis was performed with correlation distance and average-linkage method using the R package of ‘pheatmap' (version, 0.7.7).

## Results and Discussion

### Physicochemical characteristics of AMD samples

All AMD samples were characterized by low pH (1.9–2.7) and dissolved organic carbon (DOC) (2.9–6.8 mg l^-1^), and high concentrations of sulfate (6690–7931 mg l^-1^), total iron (520–2190 mg l^-1^) and heavy metals (Al, Pb, Zn, Cu, Cd, Cr and Mn) (Table 1), features typical of the acid mine environments (Johnson and Hallberg, 2003; Kuang *et al.*, 2012). The AMD environments under study were physicochemically distinct as revealed by the plots (Supplementary Figure 1a). Specifically, the DBS AMD was characterized with the lowest temperature, total iron and highest dissolved oxygen, Cu concentration, whereas FK AMD was characterized by its highest temperature, DOC, Fe^3+^, Zn and lowest pH. In comparison, the YFS and YFP samples collected from the same mine were both characterized with relatively higher total iron and Fe^2+^/Fe^3+^ ratio, indicating a lower iron oxidation rate.

### Microbial community composition and transcriptionally active taxa

Taxonomic assignment of protein-coding gene sequences in the DNA data sets revealed >100 genera in each of the four AMD communities, indicating unexpectedly high microbial diversity in these extreme environments (Kuang *et al.*, 2012), which hindered the sequence assembly at the current sequencing depth. The DBS community was dominated by archaeal Richmond Mine acidophilic nanoorganism (Baker *et al.*, 2010), *Acidithiobacillus* and *Leptospirillum* members (Figure 1b and Supplementary Figure 1b). The FK community was with high abundance of *Euryarchaeota,* including archaeal Richmond Mine acidophilic nanoorganism, *Thermoplasma* and *Ferroplasma*, likely owing to the relatively high temperature of the AMD (Table 1). The two communities from the Yunfu mine showed similarity with high abundances of *Acidithiobacillus* spp. and AMRAN (Figure 1b and Supplementary Figure 1b), likely because of their similar environmental conditions (Supplementary Figure 1a). The taxonomic assessment based on the 16S rRNA gene-bearing sequences in the DNA data sets gave generally consistent results (Figure 1a), except that no archaeal Richmond Mine acidophilic nanoorganism-associated sequences were identified. This is likely due to the lacking representation of these lesser known archaea in the Ribosomal Database Project database. Taxonomic assignment of the cDNA protein-coding gene sequences revealed that *Acidithiobacillus*, *Leptospirillum* and *Acidiphilium* were the most transcriptionally active populations in the AMD communities (Figure 1c). Specifically, *Acidithiobacillus* and *Leptospirillum* and *Leptospirillum* and *Acidiphilum* were the most active populations in the DBS and FK AMD, respectively. The YFS and YFP communities harbored active populations of *Leptospirillum* and *Acidithiobacillus*, respectively. This is unexpected, as the two AMD samples shared similar environmental conditions (Supplementary Figure 1a) and microbial compositions (Figure 1a and b). Surprisingly, archaeal Richmond Mine acidophilic nanoorganism as dominant taxa across the four AMD samples showed extremely low transcriptional activity in all communities (Figure 1b and c). However, this result was consistent with a previous metaproteomic analysis of AMD biofilms, where archaeal Richmond Mine acidophilic nanoorganism showed very low activity (Baker *et al.*, 2010), and indicated that the taxa with the highest abundances may not necessarily be the greatest contributors to the community functions (Figure 1).

### Overview of metabolic potentials and functional activities

The non-rRNA sequences-matching genes of NCBI non-redundant, extended STRING COG and KEGG databases revealed the metabolic potentials and functional activities of the AMD communities. Genes involved in energy production and conversion, translation, ribosomal structure and biogenesis, posttranslational modification, protein turnover, chaperons, amino-acid metabolism and carbohydrate metabolism dominated the transcript pools of all analyzed communities (Supplementary Table 2 and Figure 3). The dominance of these transcripts was consistent with the results from previous metaproteomic (Ram *et al.*, 2005; Bertin *et al.*, 2011) and environmental transcriptomic analyses of other AMD systems (Parro *et al.*, 2007; Moreno-Paz *et al.*, 2010), and even metatranscriptomic analyses of marine and soil ecosystems (Frias-Lopez *et al.*, 2008; Urich *et al.*, 2008), indicating the uniform high abundance of transcripts for the maintenance of basic cellular machinery, enabling growth and metabolism in the normal and extreme environments (Moran, 2009). As expected, the DBS, YFS and YFP communities exhibited more similar metabolic potentials compared with that of FK (Supplementary Figure 2c), mirroring their relatively similar environmental conditions (Supplementary Figure 1a) and community composition (Figure 1b). However, the YFS community exhibited more similar overall transcriptional profiles with that of FK (Supplementary Figures 1d and 3), which was consistent with their comparable active taxa composition (Figure 1c).

### Community-wide comparative gene transcriptional behaviors

**Figure 2:**
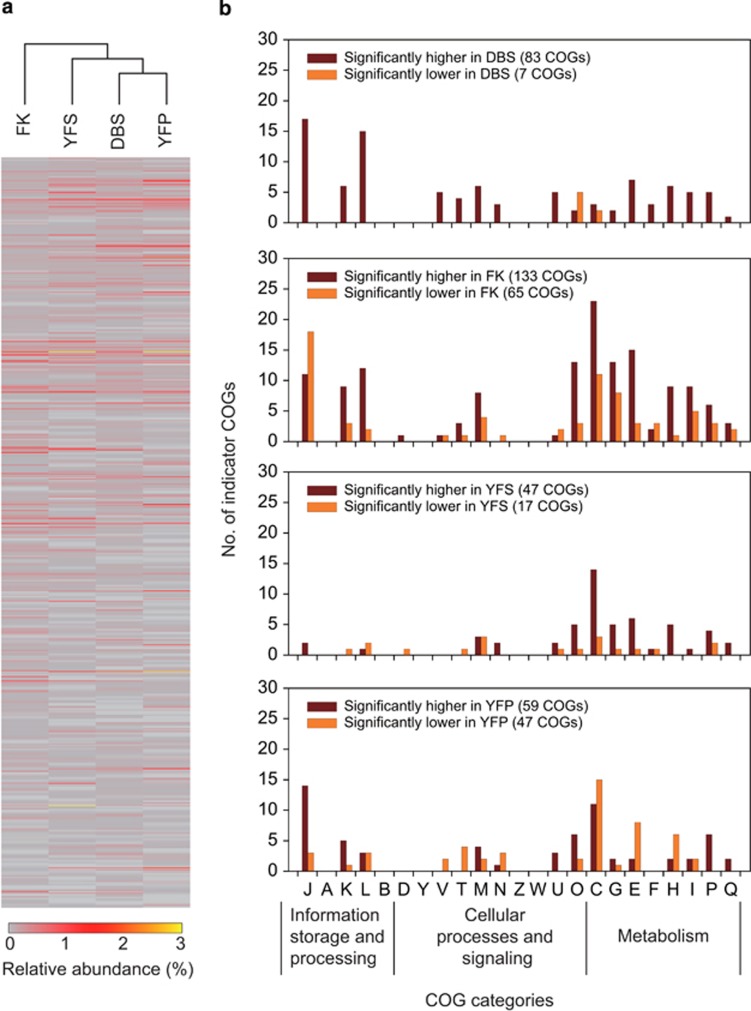
The COGs with significantly different expression levels in the four AMD communities. (**a**) Hierarchical clustering of the transcript COGs based on their relative abundances in each community. (**b**) The number and functional categories of COGs in each community with significantly higher or lower expression levels than those in the other three communities. COG categories: J: translation, ribosomal structure and biogenesis; A: RNA processing and modification; K: transcription; L: replication, recombination and repair; B: chromatin structure and dynamics; D: cell cycle control, cell division, chromosome partitioning; Y: nuclear structure; V: defense mechanisms; T: signal transduction mechanisms; M: cell wall/membrane/envelope biogenesis; N: cell motility; Z: cytoskeleton; W: extracellular structures; U: intracellular trafficking, secretion and vesicular transport; O: posttranslational modification, protein turnover, chaperones; C: energy production and conversion; G: carbohydrate transport and metabolism; E: amino-acid transport and metabolism; F: nucleotide transport and metabolism; H: coenzyme transport and metabolism; I: lipid transport and metabolism; P: inorganic ion transport and metabolism; and Q: secondary metabolites biosynthesis, transport and catabolism.

Our analysis indicated that 935 out of 2731 COGs were with significantly different expression levels across the four communities (*P*<0.05; two-tailed Fisher's exact test; Figure 2a), suggesting distinct gene transcriptional profiles. In a given community, we define a COG with a significantly higher or lower expression level than that in all other three communities as an indicator COG (of this community), which attributes the most or the fewest transcripts for the associated function across the four communities. Accordingly, 90, 198, 64 and 106 indicator COGs (higher or lower) were, respectively, identified for the communities of DBS, FK, YFS and YFP (Figure 2b and Supplementary Table 3). The FK communities harbored a large number of higher indicator COGs in posttranslational modification, protein turnover, chaperones (COG O), energy production and conversion (COG C) and amino-acid transport and metabolism (COG E) (Figure 2b), likely owing to the much higher temperature of FK AMD (43.4 ^o^C), for elevated temperature should upregulate the expression of proteins associated with these functions (Mosier *et al.*, 2014). On the basis of the functional assignment of these higher and lower indicator COGs and the dominant transcripts (see above), the associated *in situ* functional activities of the AMD communities will be discussed in the next several sections, taking into account the physicochemical differences of the four AMD environments.

#### Housekeeping functions

**Figure 3:**
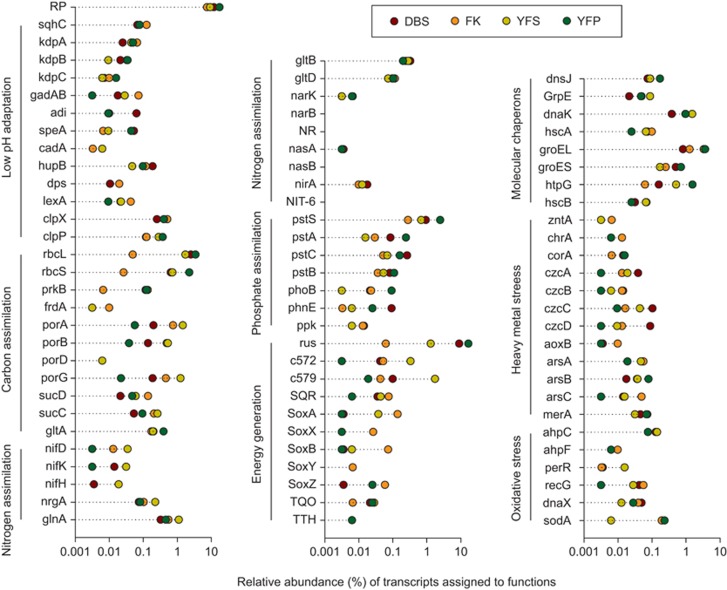
Relative abundances (%) of transcripts associated with the *in situ* functional activities of the four AMD communities, including ribosomal proteins (RP), low pH adaptation, carbon assimilation, nitrogen assimilation, phosphate assimilation, energy generation and environment stress resistance (molecular chaperons, heavy metal stress and oxidative stress). The bar showing the number of relative abundance was log scaled.

The expression levels of housekeeping function associated genes, including those for DNA-directed RNA polymerase, ribosomal proteins, elongation factors, cytochromes and ATP synthase, varied across the four communities (Supplementary Table 3). The lowest abundance of ribosomal protein transcripts (3.65% Figure 3) indicated that the microorganisms in FK community were likely with the lowest average growth rates. This speculation was supported by the fact that most of the archaeal taxa in this community showed very low activities (Figure 1b and c).

#### Low pH adaptation

AMD microorganisms are challenged to maintain a near-neutral cytoplasm and to resist the low pH (Baker-Austin and Dopson, 2007). Using a cell membrane highly impermeable to protons is an important strategy to limit the influx of protons into the cell (Konings *et al.*, 2002). The gene encoding hopanoids in bacteria for this strategy, that is, the squalene–hopene cyclase (*sqhC*) (Pearson *et al.*, 2007), was abundant in all the cDNA data sets (Figure 3). In addition, the detection of transcripts for the ABC high-affinity potassium transport system (*kdpABC*) (Figure 3) indicated that the microbes may generate an inside-positive membrane potential through active influx of K^+^ to partially deflect the inward flow of protons (Baker-Austin and Dopson, 2007). The cytoplasmic pH was also proved to be maintained by the metabolism of proton buffer molecules, like phosphate uptake (*pstSCAB*), and glutamate, arginine and lysine decarboxylation (*gadAB*, *adi*, *speA*, *cadA*) (Figure 3). Moreover, genes for other novel acid resistance factors as illustrated by Guazzaroni *et al.* (2013) in AMD environments, including the ClpXP (*clpX*, *clpP*) proteins, the transcriptional repressor LexA (*lexA*) and nucleic acid-binding proteins of Hu (*hupB*) and Dps (*dps*), were also expressed in all four communities (Figure 3). Our analyses suggested that the FK community with the lowest AMD pH (1.9) harbored the highest abundance of low pH adaptation associated transcripts that discussed above (1.14% in total), and the DBS and YFS communities with the highest pH (2.7) were, respectively, detected with *clpX* and *hupB* as a lower indicator COGs (Supplementary Table 3). These results indicated that the community-wide transcriptional behaviors for low pH adaptation were closely related to the AMD pH value, though that was not remarkably different between the AMD environment (Table 1).

#### Carbon assimilation

As typical of other AMD environments (Johnson and Hallberg, 2003), all the four AMD samples contained limited DOC (2.9–6.8 mg l^-1^; Table 1). Our analysis indicated that *Acidithiobacillus* spp. and/or *Leptospirillum* spp. were the main carbon fixers in the analyzed AMD communities, as the genes encoding the key enzymes for Calvin–Benson–Bassham cycle and reductive tricarboxylic acid cycle were highly expressed (Figure 3 and Supplementary Table 2), supporting the findings from previous transcriptomic or metaproteomic analyses of other AMD environments (Parro *et al.*, 2007; Bertin *et al.*, 2011). Notably, the collective transcriptional activities of carbon fixing genes were much lower in the FK community (Supplementary Figures 4b and c), which harbored multiple higher indicator COGs for DOC assimilation and metabolism (Supplementary Table 3), including sugar and amino-acid transporters (Poretsky *et al.*, 2010) and glucose and alcohol dehydrogenases. Our analysis indicated that the microbes in FK AMD, especially the active heterotrophic *Acidiphilium* spp. (Johnson and Hallberg, 2008), likely obtain carbon resources from the environment (DOC=6.8 mg l^-1^; Table 1).

#### Nitrogen assimilation

As reported for other AMD environment (Parro *et al.*, 2007), the concentration of nitrogen resources (for example, ammonium, nitrate, nitrite) was under detection limits (<0.05 mg l^-1^) in all analyzed AMD samples. Therefore, the knowledge of nitrogen fixation and assimilation mechanisms used by the AMD microorganisms is critical to the understanding of how they respond and adapt to the nitrogen-limited conditions (Baker and Banfield, 2003). Atmospheric N_2_, ammonium, nitrite and nitrate are important inorganic nitrogen resources for microorganisms (Arrigo, 2004). Direct observation of nitrogen fixation in low pH conditions has not been made (Baker and Banfield, 2003), and expression of the nitrogen-fixing genes were not identified in previous studies of AMD communities (Ram *et al.*, 2005; Parro *et al.*, 2007). However, our transcriptional analysis detected nitrogen-fixation transcripts (including *nifD, nifK and nifH*) in all the four communities (Figure 3), which were associated with *L. ferrooxidans* (group I), *L. ferrodiazotrophum* (group III), *At. ferrivorans* and *Acidithiobacillus* sp. GGI-221. This result indicated the key role of these taxa in nitrogen fixation of the communities, despite the relatively low abundance of the nitrogen-fixation transcripts (5–27 transcripts). The genes for ammonium transporter (*amt*) showed high transcriptional activities in all communities (Figure 3). Likewise, genes encoding the glutamine synthetase (*glnA*) and glutamate synthase (*gltBD*), which would permit the incorporation of ammonium into glutamine and then to glutamate for utilization (Leigh and Dodsworth, 2007), were also highly expressed (Figure 3), as reported by Parro *et al.* (2007) in other AMD environments. The *glnA* was identified as a higher indicator COG of YFS community (Supplementary Table 3), indicating a high need of nitrogen resources for the populated microorganism. However, the genes encoding enzymes for the utilization of nitrate and nitrite (for example, *narK, nasA, nasB, nirA, nirB*) exhibited very low transcriptional activities (Figure 3). Together, these results indicated that the AMD communities likely obtained nitrogen resources via nitrogen fixation and ammonium assimilation. This scenario is reasonable, as the analyzed samples were from open AMD environments where external ammonium inputs are highly possible.

#### Phosphate assimilation

Phosphate is vital for the synthesis of many biomacromolecules (Lamarche *et al.*, 2008), and could also help to deal with the low pH stress via cytoplasmic buffering of H^+^ (Baker-Austin and Dopson, 2007). The genes of phosphate transport system (*pstSCAB*) were highly abundant in our cDNA data sets (Figure 3), which were with the transcriptionally active taxa of *Acidithiobacillus*, *Leptospirillum* and *Acidiphilium* and other less active taxa. These results indicated the ubiquitous behavior of phosphate uptake for the AMD microorganisms, especially the active taxa, which was supported by the transcription of the putative phosphate regulon response regulator gene of *phoB* in them (Figure 3; Lamarche *et al.*, 2008). Furthermore, previous studies have found that the expression of these genes seemed to be upregulated under phosphate starvation for AMD microorganisms like *Acidithiobacillus* and *Leptospirillum* spp. (Vera *et al.*, 2003; Parro *et al.*, 2007). Although the concentration of phosphate could not be determined in our AMD environments because of its low content, it is reasonable to speculate (based on the high expression of *pstSCAB and phoB*) that the AMD communities, especially that of YFP, which exhibited the highest expression levels of these genes (Figure 3; *pstS* and *pstC* were higher indicator COGs of YFP, Supplementary Table 3), may encounter a phosphate limited condition. The coprecipitation of phosphate with Fe^3+^ and Al^3+^ (Grzmil and Wronkowski, 2006), which were also with high concentrations in the YFP AMD (Table 1), may partially account for this scenario. Meanwhile, the transcripts for transporters of phosphonate and polyphosphate were also detected (*phnE* and *ppk*; Figure 3), indicating multiple strategies for phosphorus resources and further supporting the speculated phosphate limited condition.

#### Energy generation

Energy generation is crucial for microorganisms to drive physiologically important processes and thus survive in extreme environments (Tyson *et al.*, 2004). Genes for ATPase (F-type), which makes use of pH gradient (pH is typically 10^4^–10^6^ times higher outside of the cell in AMD) to uptake protons to generate ATP (Johnson and Hallberg, 2008), were abundantly detected in the cDNA data sets (Figure 3 and Supplementary Table 2). The obtained protons are usually coupled with O_2_ to generate H_2_O with the consumption of electrons from ferrous iron (Fe^2+^) and/or sulfur oxidation (Bird *et al.*, 2011). Our data showed that genes encoding the key proteins for Fe^2+^ oxidation, including the rusticyanin of *Acidithiobacillus* spp. (Valdés *et al.*, 2008) and cytochrome 572 and cytochrome 579 of *Leptospirillum* spp. (Ram *et al.*, 2005; Goltsman *et al.*, 2009), were highly expressed (Figure 3 and Supplementary Table 2), indicating that these active populations may generate energy through iron oxidation in the AMD communities. The FK community contributed the fewest transcripts in Fe^2+^ oxidation, the low availability of Fe^2+^ (characterized by low Fe^2+^/Fe^3+^ ratio; Table 1) and dissolved oxygen of the FK AMD may partially account for this scenario (Parro *et al.*, 2007). Also, multiple sulfur oxidation transcripts were detected (Figure 3), including those for sulfur oxidation multienzyme complex, sulfide:quinone oxidoreductase, thiosulfate:quinone oxidoreductase, and tetrathionate hydrolase, indicating the activity of sulfur oxidizing-based energy metabolisms. Notably, our analyses showed that sulfur oxidation was mainly executed by the less active taxa of *At. thiooxidans* and *At. caldus*, although the active taxa of *At. ferrivorans* and *L. ferrodiazotrophum* may also execute this function.

#### Environmental stress

Molecular chaperones are capable of helping with protein folding and refolding for stress resistance, multiple associated transcripts were detected with high abundances (Figure 3 and Supplementary Table 2). The DBS community harbored three lower indicator COGs for chaperons, whereas the FK, YFS and YFP communities were detected with 1, 3 and 2 chaperones associated higher indicator COGs (Supplementary Table 3), likely indicating a relatively benign environment of the DBS AMD compared with the other three. However, the DBS community harbored the most transcripts for heavy metal resistance, including those for Cd, Cr, Zn, Co, As, Ag, Mn and Hg (Figure 3). Notably, DBS community was detected with three higher indicator COGs (*czcA, czcB, czcD*; Figure 3), which were responsible for Co^2+^/Zn^2+^/Cd^2+^ efflux (Supplementary Table 3), this may be due to the high concentrations of Zn and Cd in DBS AMD (Table 1). Moreover, genes involved in defense against oxidative stress were also highly expressed (Figure 3), including those encoding the peroxiredoxin (*ahpC*), superoxide dismutase (*sodA*) and hydroperoxide reductase (*dnaX*), with the majority of which was associated with the active taxa (Figure 1c), supporting their ecological success in the AMD. Collectively, our analyses thus confirmed the diverse stress resistance mechanisms that is used by AMD microorganisms to survive and thrive in the extreme environments (Tyson *et al.*, 2004; Bertin *et al.*, 2011).

#### Other functional activities

Four and six transposase associated with higher indicator COGs were, respectively, detected in the communities of DBS and FK, where one lower indicator COG for transposase was identified in both YFS and YFP communities (Supplementary Table 3). This result indicated higher activities of the former communities in genomic material rearrangement for environmental adaptation. In addition, the DBS community harbored one higher indicator COG for *S*-adenosylmethionine decarboxylase associated with *At. ferrivorans* and *At. ferrooxidans*, two for pilus assembly proteins associated with *At. ferrivorans*, and two for type II and III secretion system proteins associated with *At. ferrooxidans* (Supplementary Table 3), indicating a highly active behavior of quorum sensing and biofilm formation (Lee *et al.*, 2009; Yelton *et al.*, 2013) for microorganisms in this environment.

### Organism-wide comparative gene transcriptional behaviors

The phylogenetically divergent taxa in natural communities may exhibit distinct transcriptional profiles in response and adaptation to different biotic or abiotic factors (Parro *et al.*, 2007; Moreno-Paz *et al.*, 2010; Liu *et al.*, 2011; Mueller *et al.*, 2011; Steward *et al.*, 2012). Hierarchical clustering analysis of the top three most active taxa in each AMD community (Supplementary Figure 4) based on their DNA and cDNA COGs abundances showed that the same taxa harbored similar metabolic potentials in different communities as expected but generally exhibited varying transcriptional behaviors (Supplementary Figure 5), indicating distinct response and adaptation mechanisms under different environmental conditions (Table 1). To further reveal this, detailed analyses were conducted for the organisms of *At. ferrivorans* and *L. ferrodiazotrophum*, both of which were among the most active taxa in three of the four AMD communities (Supplementary Figure 4).

**Figure 4:**
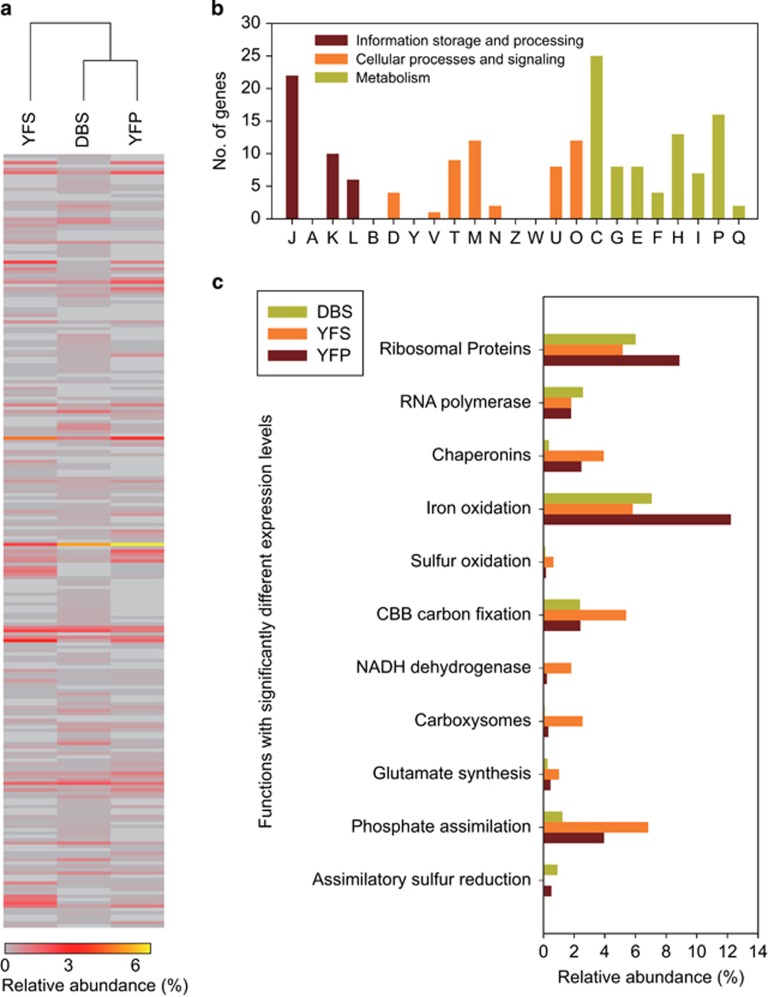
Comparative transcriptional activities of *At. ferrivoran*s in the DBS, YFS and YFP communities. (**a**) Clustering analysis based on the 211 genes with significantly different expression levels (*P*<0.05; two-tailed fisher exact test), indicating *in situ* functions of *At. ferrivorans* in DBS and YFP were more similar. (**b**) Functional assignment of the significant transcripts based on COG categories. (**c**) The key functional activities that varied for *At. ferrivorans* in the three communities. See Supplementary Table 4 for detailed information of the transcripts for these functions.

*Case study of* At. ferrivorans. The psychrotolerant acidophile *At. ferrivorans* (Hallberg *et al.*, 2010) was highly active in DBS, YFS and YFP (Supplementary Figure 4) but with low abundance and activity in FK, likely due to the high solution temperature (43.3 ^o^C; Table 1). Our analyses detected 211 genes (1831 in total) of *At. ferrivorans* with significantly different transcriptional levels across the three communities (*P*<0.05, two-tailed Fisher's exact test), although those in DBS and YFP exhibited generally more similar activities (Figure 4a). Functional analysis of these transcripts indicated that *At. Ferrivorans* in these communities exhibited different activities spanning a broad range of functions.

Thirty eight significantly different transcripts were associated with information storage and processing, especially those for translation, ribosomal structure and biogenesis (COG J; Figure 4b). These transcripts mainly were for ribosomal proteins, which could indicate the high growth rates (Gifford *et al.*, 2013) of *At. ferrivorans* in these communities as was further supported by the high expression of genes for RNA polymerase (Figure 4c). Forty eight significant transcripts were involved in cellular processes and signaling (Figure 4b). Notably, the three genes for chaperonins of GroEL (Hsp60), dnaK and htpG (Hsp90) all exhibited a significant upregulation in YFS and YFP (Figure 4c). This was likely due to the much higher temperatures of these two samples compared with DBS (Table 1), as these chaperonins are well-known proteins, responding to heat-shock (Mogk *et al.*, 2003), although they could also deal with other environmental stresses.

The majority of the significantly different transcripts (106 genes; Figure 4b) were associated with metabolism, especially for energy production and conversion (COG C; 25 transcripts). *At. Ferrivorans* could generate energy via both iron and sulfur oxidation (Hallberg *et al.*, 2010). Twelve out of the 14 genes in the *rus* operon for iron oxidation (Liljeqvist *et al.*, 2013) and nine genes for sulfur oxidation were expressed. Interestingly, *At. ferrivorans* in YFS contributed more transcripts for sulfur oxidation, and fewer for iron oxidation, compared with that in DBS and YFP (Figure 4c). This may be associated with the carbon-fixation activity (via the Calvin–Benson–Bassham pathway), which was most active for *At. ferrivorans* in YFS. This activity requires electrons for the reduction of NAD^+^ to NADH (Johnson and Hallberg, 2008), the significant upregulation of carbon fixing genes in *At. ferrivorans* of YFS was supported by the significant upregulation of genes encoding NADH dehydrogenase complex, and sulfur oxidation is a more effective strategy to provide electrons compared with Fe^2+^ oxidation; on the other hand, carboxysomes may be produced in *At. ferrivorans* of YFS, as indicated by the significant higher expression of involved genes, suggesting a carbon-limiting condition, as reported in other *Acidithiobacillus* spp. (Shively *et al.*, 1998); moreover, it seemed that the more energetic substrate of sulfur tended to need for more cellular carbon during growth, as suggested for *At. ferrooxidans* (Appia-Ayme *et al.*, 2006). *At. ferrivorans* in all three communities could fix nitrogen, whereas that in YFS showed significantly higher activities of glutamine synthetase and glutamate synthase (Figure 4c), indicating a much need for nitrogen resources. In addition, *At. ferrivorans* in all three communities (especially in YFS) showed high activities in the uptake of phosphate. Moreover, *At. ferrivorans* in DBS and YFP may execute assimilatory sulfur reduction for the utility of sulfur as indicated by the upregulation of sulfite reductase genes of *cysI*, *cysJ* and *cysH*. Collectively, these lines of evidence indicate that *At. ferrivorans* in YFS contributed more for carbon, nitrogen and phosphate assimilation, whereas those in DBS and YFP were likely active in sulfur assimilation.

**Figure 5:**
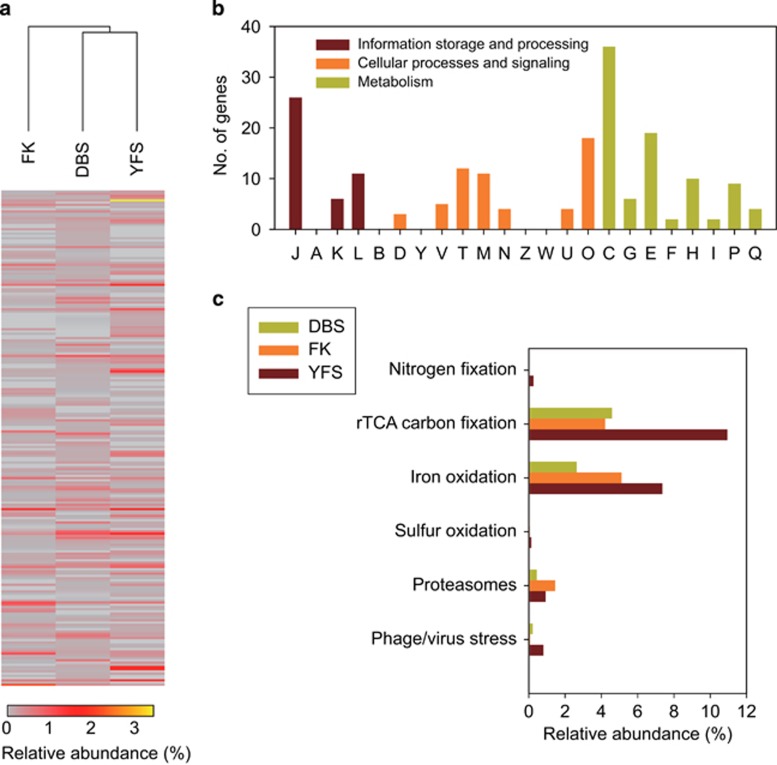
Comparative transcriptional activities of *L. ferrodiazotrophum* in the DBS, FK and YFS communities. (**a**) Clustering analysis based on the 223 genes with significantly different expression levels (*P*<0.05; two-tailed fisher exact test), indicating *in situ* functions of *At. ferrivorans* in DBS and YFS were more similar. (**b**) Functional assignment of the significant transcripts based on COG categories. (**c**) The key functional activities that varied for *L. ferrodiazotrophum* in the three communities. See Supplementary Table 5 for detailed information of the transcripts for these functions.

*Case study of* L. ferrodiazotrophum. The recently characterized iron-oxidizing and free-living nitrogen fixer *L. ferrodiazotrophum* (Tyson *et al.*, 2005) represented >5% of the total transcripts of the DBS, FK and YFS communities (Supplementary Figure 4). A total of 1997 transcripts were identified, 223 of which exhibited significantly different expression levels among the three communities (Figure 5a).

Similar to those of *At. ferrivorans*, the majority of these transcripts (117 transcripts; Figure 5b) were associated with metabolism-related functions. *L. ferrodiazotrophum* was considered as a keystone species in the Richmond Mine AMD ecosystems for the ability of nitrogen fixation (Tyson *et al.*, 2004; 2005); however, no proteins involved in nitrogen fixation were detected in previous metaproteomics studies (Ram *et al.*, 2005; Goltsman *et al.*, 2009). Seven of the 16 genes of the *nif* operon of *L. ferrodiazotrophum* (Tyson *et al.*, 2005) were expressed in the three AMD communities (Figure 5c), with a total of 17 transcripts. *L. ferrodiazotrophum* in YFS contributed the most in nitrogen fixation, with >10 times of associated transcripts in abundance (0.26%) compared with those in DBS and FK. For the carbon-fixation activity, all genes of the reductive tricarboxylic acid cycle in *L. ferrodiazotrophum* (Goltsman *et al.*, 2009) were expressed (Figure 5c), and those in YFS accounted for a total abundance of 10.9%, as compared with 4.6% and 4.2% for those in DBS and FK (Supplementary Table 5). The genes for Fe^2+^ and sulfur oxidation (that is, sulfide-quinone reductase) were also detected, with the highest expression levels in YFS. These results suggest that this organism in YFS contributed much in the fixation of carbon and nitrogen via Fe^2+^ and sulfur oxidation, as *At. ferrivorans* did in the same community (see above). We further identified the genes for proteasome, which collectively accounted for a transcript abundance of 0.44%, 1.45% and 0.93% in DBS, FK and YFS, respectively (Supplementary Table 5). Within the Bacteria, proteasome has only been found in actinobacteria (De Mot, 2007) and *Leptospirillum* group II and III (Goltsman *et al.*, 2009), and this molecular machine is involved in defence against environmental stress via degrading the unneeded or damaged proteins (De Mot *et al.*, 2007). It is reasonable to speculate that the degraded proteins could be further utilized (as organic nitrogen resources) by both *L. ferrodiazotrophum* and other microorganisms in the AMD communities. Also detected for *L. ferrodiazotrophum* in all three communities were several transcripts involved in stress response to phage or virus, including those for clustered regularly interspaced short palindromic repeats associated (Cas) proteins and phage integrase proteins (Supplementary Table 5), implying the biotic stress in the extreme AMD environments.

## Conclusions and perspectives

Our comparative metagenomic and metatranscriptomic analyses have provided a detailed gene transcriptional blueprint for the naturally occurring, low diversity microbial communities in the extreme AMD environments, demonstrating how these extraordinary microorganisms respond and adapt to the different physicochemical conditions at both community- and organism-level. The transcriptional analyses of dominant transcripts and indicator COGs revealed a high diversity of transcripts harbored by these acidophilic assemblages. These transcripts were associated with a wide range of functions, the expression levels of which were closely related to the physicochemical characteristics of the AMD systems. In particular, the detection of nitrogen-fixation transcripts provided clues for nitrogen-fixing activities in AMD communities. Detailed analysis of the transcriptional behaviors of the most active organisms revealed differential gene expression patterns, likely reflecting their distinct roles and life state in different communities. For example, both *At. ferrivorans* and *L. ferrodiazotrophum* in YFS contributed more transcripts for carbon and nitrogen assimilation than their counterparts in other communities. The ecological significance of less active taxa, including their irreplaceable role for the whole-community function as well as their contribution in community assembly and dynamics, has been documented in marine environment (Musat *et al.*, 2008; Galand *et al.*, 2009). However, with the current sequencing depth of 454 pyrosequencing and the inefficient removal of rRNA sequences from total RNA, we failed to obtain sufficient transcriptional information for the less active populations in the AMD communities, hindering the understanding of their response and adaptation mechanisms at gene transcription level. With the benefit of more efficient and unbiased rRNA-depletion approaches (He *et al.*, 2010) and the development of higher-throughput sequencing technologies (for example, Illumina HiSeq, ABI Solid), future studies could be designed to explore gene transcription profiles along environmental gradients or through time series for both dominant and rare taxa, and to reveal the transcriptional dynamics of microbial communities in response and adaptation to the changing environmental conditions or associated with sequential development stages.

## Figures and Tables

**Table 1:** Physicochemical characteristics of the four AMD samples

*Sample*	*pH*	*Temperature*	*Concentration (mg* *l*^-1^)												
		*(°C)*	*DO*	*DOC*	*T-Fe*	*Fe*^*2+*^	*Fe*^*3+*^	*SO*_*4*_^*2-*^	*Al*	*Pb*	*Zn*	*Cu*	*Cd*	*Cr*	*Mn*
DBS	2.7	21.4	5.00	3.50	520	100	420	6966	168	0.19	80.1	60.36	0.40	0.11	116.7
FK	1.9	43.3	1.10	6.80	1240	210	1030	6690	53	0.23	144.5	4.35	0.25	0.39	13.7
YFS	2.7	31.8	3.80	2.90	2060	1090	980	6883	117	0.13	4.9	0.06	ND	0.21	27.7
YFP	2.5	32.6	1.00	3.90	2230	1350	880	7931	1878	0.40	80.9	0.02	0.01	0.23	145.7

Abbreviations: DBS, Dabaoshan; DO, dissolved oxygen; DOC, dissolved organic carbon; FK, Fankou; ND, not detected; T-Fe, total iron; YFP, Yunfu pond; YFS, Yunfu stream.
