# Supplementary material for: Comparative metagenomic and metatranscriptomic analyses of microbial communities in acid mine drainage
Source: ISME J. 2014 Dec 23;9(7):1579–92. doi: 10.1038/ismej.2014.245 (PMC4478699; doi:10.1038/ismej.2014.245)
Supplement: Supplementary Information [file ismej2014245x1.doc]

**Supplementary Information**

**for**

**Comparative metagenomic and metatranscriptomic analyses of microbial communities**

**in acid mine drainage**

Lin-xing Chen^1^, Min Hu^1^, Li-nan Huang, Zheng-shuang Hua, Jia-liang Kuang, Sheng-jin Li and Wen-sheng Shu

*State Key Laboratory of Biocontrol, Key Laboratory of Biodiversity Dynamics and Conservation of Guangdong Higher Education Institutes, College of Ecology and Evolution, Sun Yat-sen University, Guangzhou 510275, PR China*

Correspondence: Wen-sheng Shu, College of Ecology and Evolution, Sun Yat-sen University, Guangzhou 510275, PR China.

Tel.: +86 20 39332933

Fax: +86 20 39332944

E-mail: [shuws@mail.sysu.edu.cn](mailto:shuws@mail.sysu.edu.cn)

^1^ These authors contributed equally to this work.

**Supplementary methods**

*Sampling procedures*

The acid mine drainage (AMD) samples were collected and filtered on site. Briefly, for each sample, four replicates of 5 L AMD samples (20 L in total) were pre-filtered through 1.6 μm GF/A filters (149 mm diameter, Whatman) on to 0.22 μm PES filters (149 mm diameter, PALL) using a two-headed peristaltic pump system. The 0.22 μm filters were immediately transferred to sterilized tubes and frozen in liquid nitrogen before being transported to the laboratory where they were stored at -80 °C before DNA/RNA extraction.

*Physicochemical analyses*

The physicochemical characteristics of the AMD samples were determined as previously described (Kuang *et al*., 2013). Briefly, temperature, pH and dissolved oxygen (DO) were measured on site using specific electrodes. The concentrations of ferrous iron (Fe^2+^) and ferric iron (Fe^3+^) were determined by UV colorimetric assay with 1,10-phenanthroline at 530 nm (Hill *et al*., 1978). Concentrations of sulfate (SO_4_^2-^) were determined by a BaSO_4_-based turbidimetric method (Chesnin and Yien, 1951). Heavy metals (including total Fe, Al, Pb, Zn, Cu, Cd, Cr and Mn) were measured by inductively-coupled plasma optical emission spectrometry (ICP-OES; Optima 2100DV; Perkin-Elmer, Massachusetts, USA).

*DNA extraction*

Community genomic DNA was extracted from the filters as described previously (Fuhrman *et al.*, 1988). 3 ml of lysis buffer (100 mM NaCl, 10 mM Tris, 1 mM EDTA) with lysozyme (5 mg/ml) was added to the 0.22 μm PES filter upon thawing, followed by vortexing for 10 min to lyse attached cells with beads from the Mo-Bio DNA PowerSoil kit (Carlsbad, CA). After incubating at 37 °C for 30 min, proteinase K (0.5 mg/ml) and SDS (1%) were added into the filters, and incubated at 55°C for 20 min, followed by a further incubation at 70 °C for 5 min. Lysate was extracted twice with phenol:chloroform:IAA (25:24:1) and once with chloroform:isoamyl alcohol (24:1) and then precipitated with ethanol. DNA was collected by centrifugation, resuspended in TE buffer and subjected to a final clean-up step (QIAamp mini spin columns, Qiagen).

*RNA extraction, rRNA subtraction, RNA amplification and cDNA synthesis*

Total RNA was extracted using a modified version of the *mir*Vana^TM^ RNA isolation kit (Ambion, Austin, TX, USA). Frozen samples were first thawed in Lysis/Binding buffer (Ambion), followed by vortexing for 10 min to lyse attached cells with RNase-free beads from the Mo-Bio RNA PowerSoil kit (Carlsbad, CA). This step was followed by centrifugation at 7500 g for 5 min, then RNA was isolated from the supernatant with the *mir*Vana^TM^ RNA Isolation kit according to the manufacturer’s instructions. The residual DNA was removed using the Turbo DNA-free kit (Ambion) and the RNA was purified using an RNeasy MinElute Cleanup kit (Qiagen). The bacterial and archaeal 16S and 23S ribosomal RNA (rRNA) transcripts in total RNA samples were reduced as previously described (Stewart *et al.*, 2010). The rRNA-depleted RNA was purified with an RNA purification kit (Qiagen). The purified RNA samples were amplified with the MessageAmp II-Bacteria kit (Ambion) as described previously (Frias-Lopez *et al*., 2008). The amplified RNA was then converted to cDNA using the SuperScript III First Strand Synthesis System (Invitrogen) with random hexamer primers, and the second-strand was synthesized with the SuperScript Double-Stranded RNA synthesis kit (Invitrogen). The cDNA so obtained was purified using the QIAquick PCR purification kit (Qiagen) to remove residual reactants and nucleotides, digested with BpmI (37 °C, 5 h) to removed poly(A) tails, and then purified again using the QIAquick PCR purification kit (Qiagen).

*Bioinformatics analyses*

The identification of rRNA gene sequences. The rRNA reads in DNA and cDNA datasets were identified using BLASTn, against a combined 5S, 16S, 18S, 23S, and 25S rRNA database from the ARB LSU and SSU databases ([www.arb-silva.de](http://www.arb-silva.de/); Pruesse *et al.*, 2007), and those aligned with a bit score ≥ 50 were identified as rRNA sequences according to Gifford *et al*. (2010).

Taxonomic classification of 16S rRNA gene sequences. Within the rRNA sequences, the 16S rRNA sequences were identified by BLASTn search against the Ribosomal Database Project (RDP) database (release 10; Cole *et al.*, 2009), with the criteria as follows: a bit score ≥ 50, query alignment length ≥ 100 bp and alignment coverage ≥ 80% (according to Shi *et al*., 2011). The 16S rRNA sequences identified were further assigned to phylogenetical groups using the RDP Classifier (Wang *et al.*, 2007) with a minimum confidence of 50%. The relative abundance of a given taxon was calculated as a percentage of the number of 16S rRNA sequences assigned to this taxon, dividing by the total number of sequences assigned to all taxa.

The identification and removal of artificial replicates. It is widely reported that 454 pyrosequencing sequences contain a large number of artificial replicates, which may lead to over-estimation of the abundance of species and genes (Gomez-Alvarez *et al.*, 2009; Stewart *et al.*, 2010). For each of the AMD DNA and cDNA non-rRNA datasets, the pyrosequencing artificial replicates (sequences sharing 100% nucleotide identity and length) were identified and removed with CD-Hit (Li and Godzik, 2006), with the parameters set as –c = 1, -aL = 1, -aS = 1. The output *.fasta files represented the non-replicate datasets, and were used for further analysis.

Taxonomic classification of protein-coding gene sequences. The non-rRNA, non-replicate DNA and cDNA sequences were compared against the National Center for Biotechnology Information non-redundant protein database (NCBI-nr) using BLASTx (bit score ≥ 40). The taxonomic information of the protein-coding gene sequences was obtained by parsing the NCBI-nr comparison results using the lowest common ancestor algorithm in MEGAN (Huson *et al*., 2007), with the default parameters. Based on the goals of this study, the sequences assigned to bacteria and archaea were extracted for further analysis. The relative abundance of a given taxon was calculated as a percentage of the number of sequences assigned to this taxon dividing by the total number of sequences assigned to all taxa.

Functional annotation. The functional information of these sequences was firstly obtained from the matching genes in the NCBI-nr database. To further reveal the function, the protein-coding sequences were assigned to the Clusters of Orthologous Groups of proteins (COGs) in the extended COG database (STRING; Franceschini *et al*., 2013), and also to the Kyoto Encyclopedia of Genes and Genomes (KEGG) database, using BLASTx with an threshold of bit scores ≥ 40. The top blast hit was used as annotation, then the sequences were assigned to COG functional categories and KEGG pathways.

**Supplementary references**

Chesnin L, Yien CH. (1951). Turbidimetric determination of available sulphates. *Proc Soil Sci Soc Am* **15**: 149-151.

Cole JR, Wang Q, Cardenas E, Fish J, Chai B, Farris RJ *et al*. (2009). The Ribosomal Database Project: improved alignments and new tools for rRNA analysis. *Nucleic Acids Res* **37**: d141–d145.

Fuhrman JA, Comeau DE, Hagström Å, Chan AM. (1988). Extraction from natural planktonic microorganisms of DNA suitable for molecular biological studies. *Appl Environ Microbiol* **54**: 1426-1429.

Gifford SM, Sharma S, Rinta-Kanto JM, Moran MA (2010). Quantitative analysis of a deeply sequenced marine microbial metatranscriptome. *ISME J* **5**: 461–472.

Gomez-Alvarez V, Teal TK, Schmidt TM. (2009). Systematic artifacts in metagenomes from complex microbial communities. *ISME J* **3**: 1314–1317.

Hill AG, Bishop E, Coles LE, McLaughlan EJ, Meddle DW, Pater MJ *et al*. (1978). Standardized general method for the determination of iron with 1,10-phenanthroline. *Analyst* **103**: 391-396.

Huson DH, Auch AF, Qi J, Schuster SC. (2007). MEGAN analysis of metagenomic data. *Genome Res* **17**: 377–386.

Shi Y, Tyson GW, Eppley JM, Delong EF. (2011). Integrated metatranscriptomic and metagenomic analyses of stratified microbial assemblages in the open ocean. ISME J 5: 999–1013.

Stewart FJ, Ottesen EA, DeLong EF. (2010). Development and quantitative analyses of a universal rRNA-subtraction protocol for microbial metatranscriptomics. *ISME J* **4**: 896–907.

Wang Q, Garrity GM, Tiedje JM, Cole JR. (2007). Naive Bayesian classifier for rapid assignment of rRNA sequences into the new bacterial taxonomy. *Appl Environ Microbiol* **73**: 5261-5267.

**
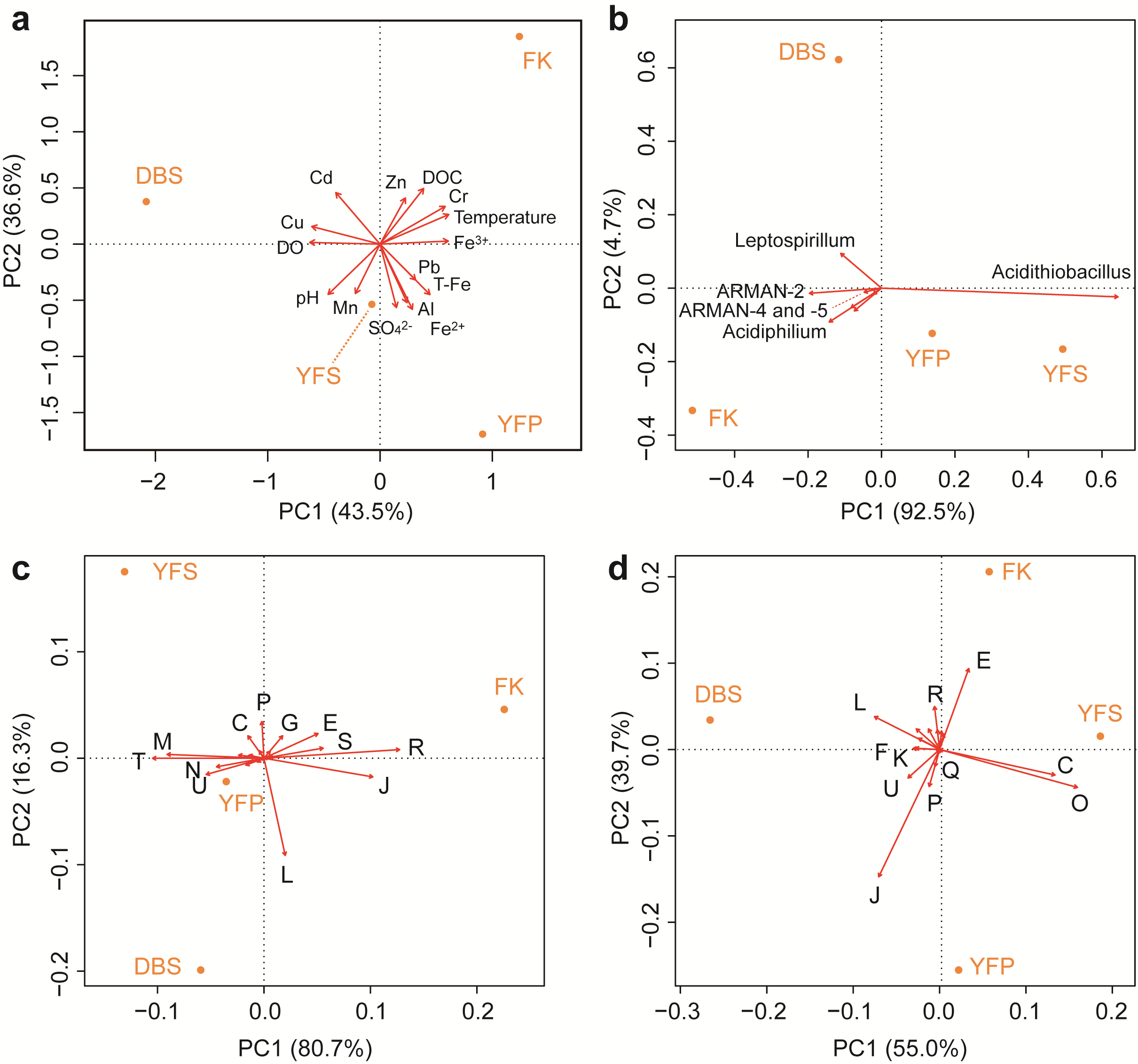
**

**Figure S1** Principal Component Analysis of the four AMD based on their (a) physicochemical characteristics, (b) community composition, (c) metabolic potentials, and (d) functional activities. Physicochemical characteristics as listed in Table 1 of Main text were z (0,1) normalized. Microbial composition, and COG functional categories based metabolic potentials and functional activities are given in relative abundance (%). COG categories: J: Translation, ribosomal structure and biogenesis; K: Transcription; L: Replication, recombination and repair; T: Signal transduction mechanisms; M: Cell wall/membrane/envelope biogenesis; N: Cell motility; U: Intracellular trafficking, secretion, and vesicular transport; O: Posttranslational modification, protein turnover, chaperones; C: Energy production and conversion; G: Carbohydrate transport and metabolism; E: Amino acid transport and metabolism; F: Nucleotide transport and metabolism; P: Inorganic ion transport and metabolism; and Q: Secondary metabolites biosynthesis, transport and catabolism.

**
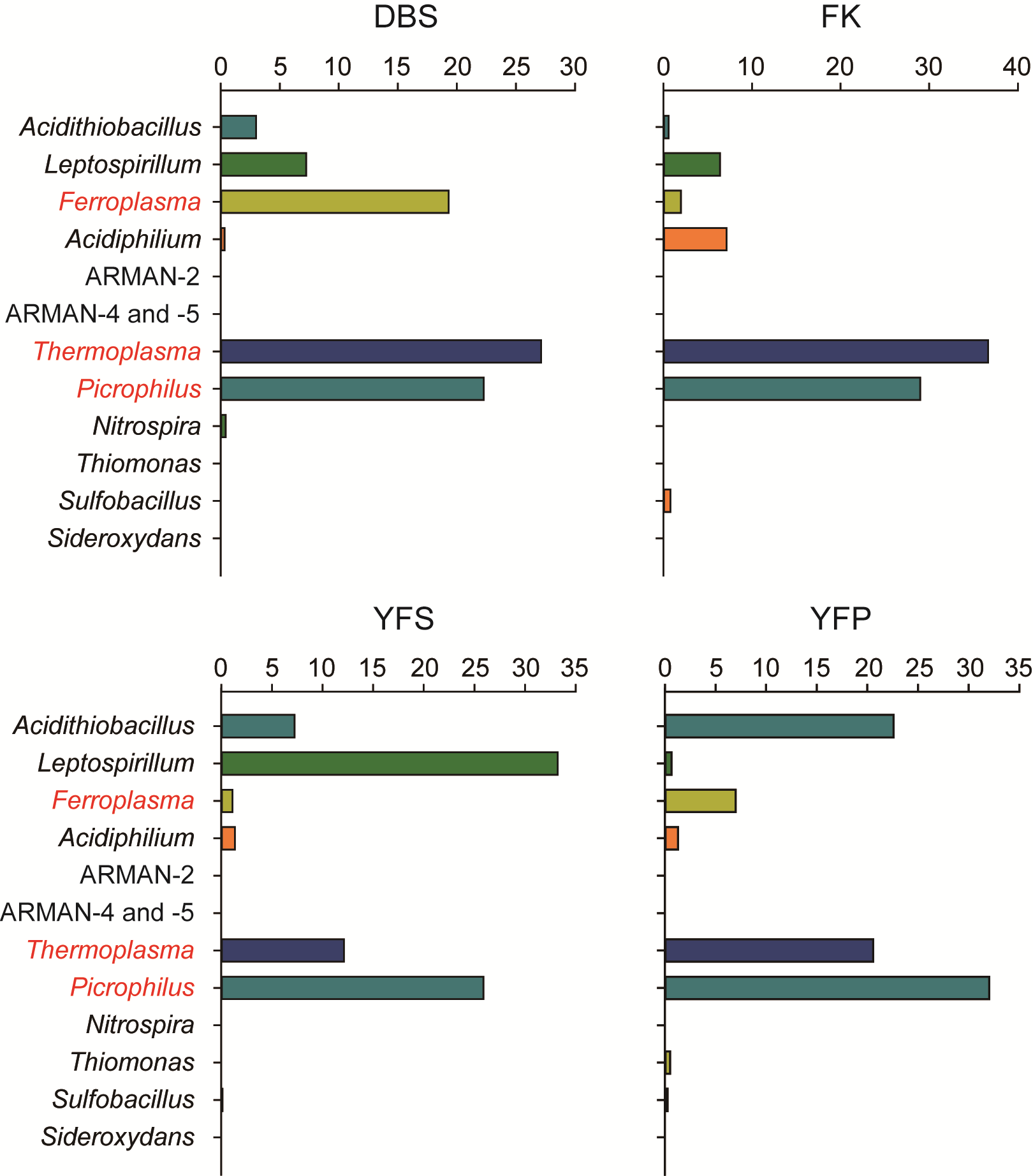
**

**Figure S2** 16S rRNA gene bearing RNA sequences assigned to microbial taxa for the four AMD communities. This Figure indicates that the removal of rRNA sequences during the experimental processes seems to be insufficient for some archaeal taxa (genus names in red) and dominant taxa (i.e., *Acidithiobacillus*, *Leptospirillum*, *Acidiphilium*). Refer to main text for sample abbreviations.


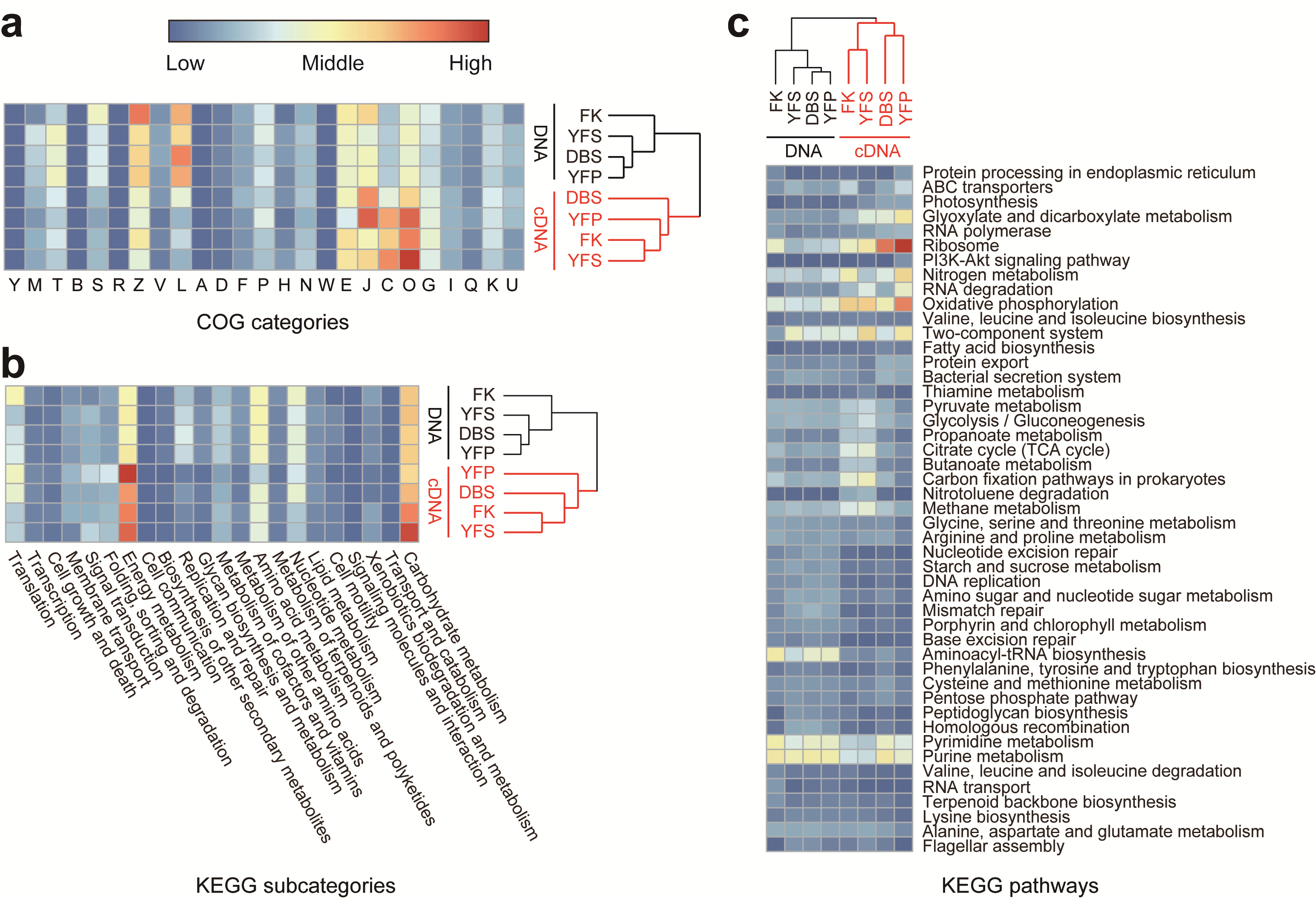


**Figure S3** Functional clustering analyses of the DNA and cDNA datasets of the four AMD samples. Hierarchical clustering based on their relative abundance of protein-coding gene sequences assigned to (a) COG categories, (b) KEGG subcategories and (c) KEGG pathways (relative abundance > 1%), was performed with correlation distance and average-linkage method with the R package of ‘pheatmap’.


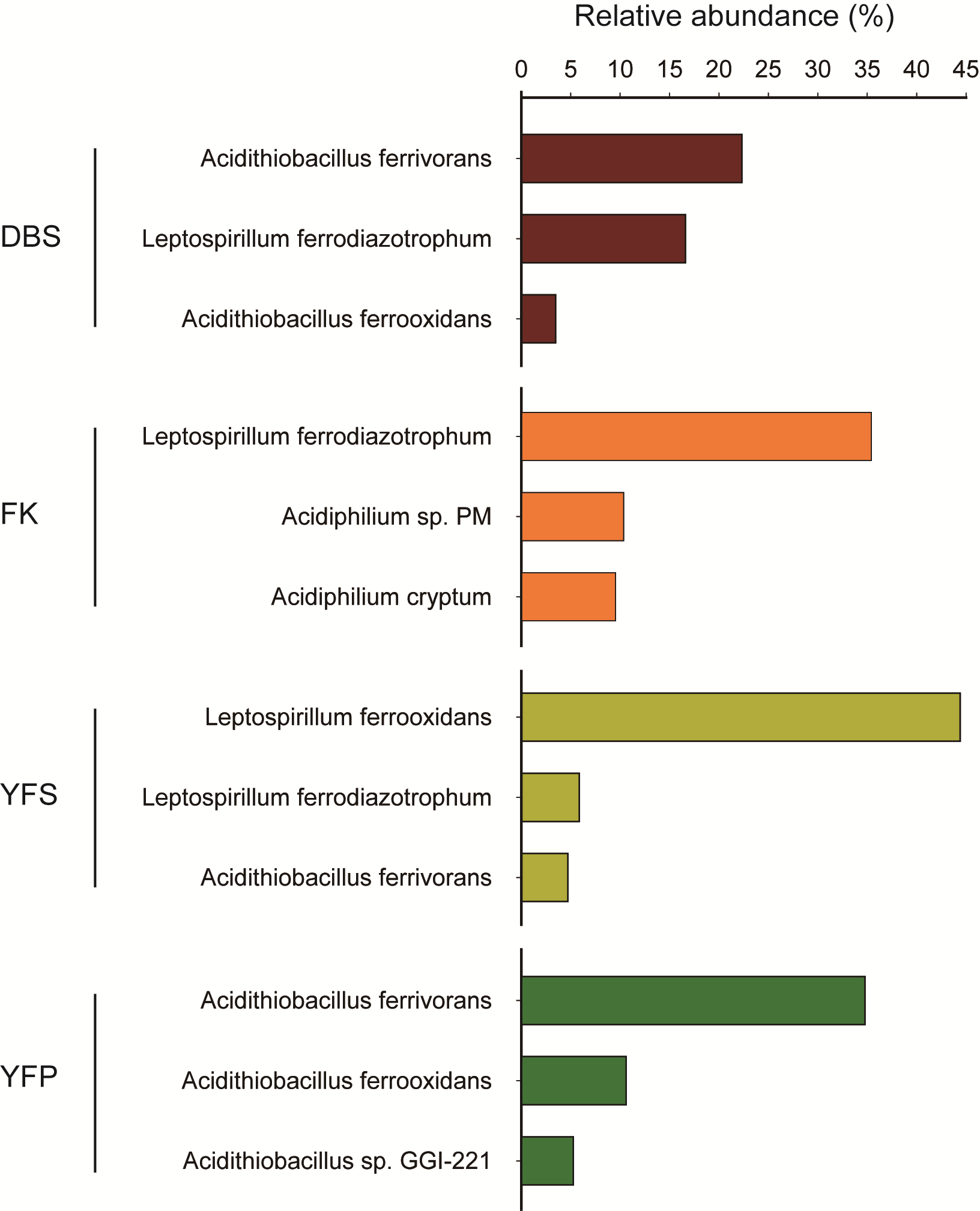


**Figure S4** The relative transcriptional contribution of the top 3 most active taxa in each of the four AMD communities. The relative abundance of transcripts assigned to each taxon are shown.


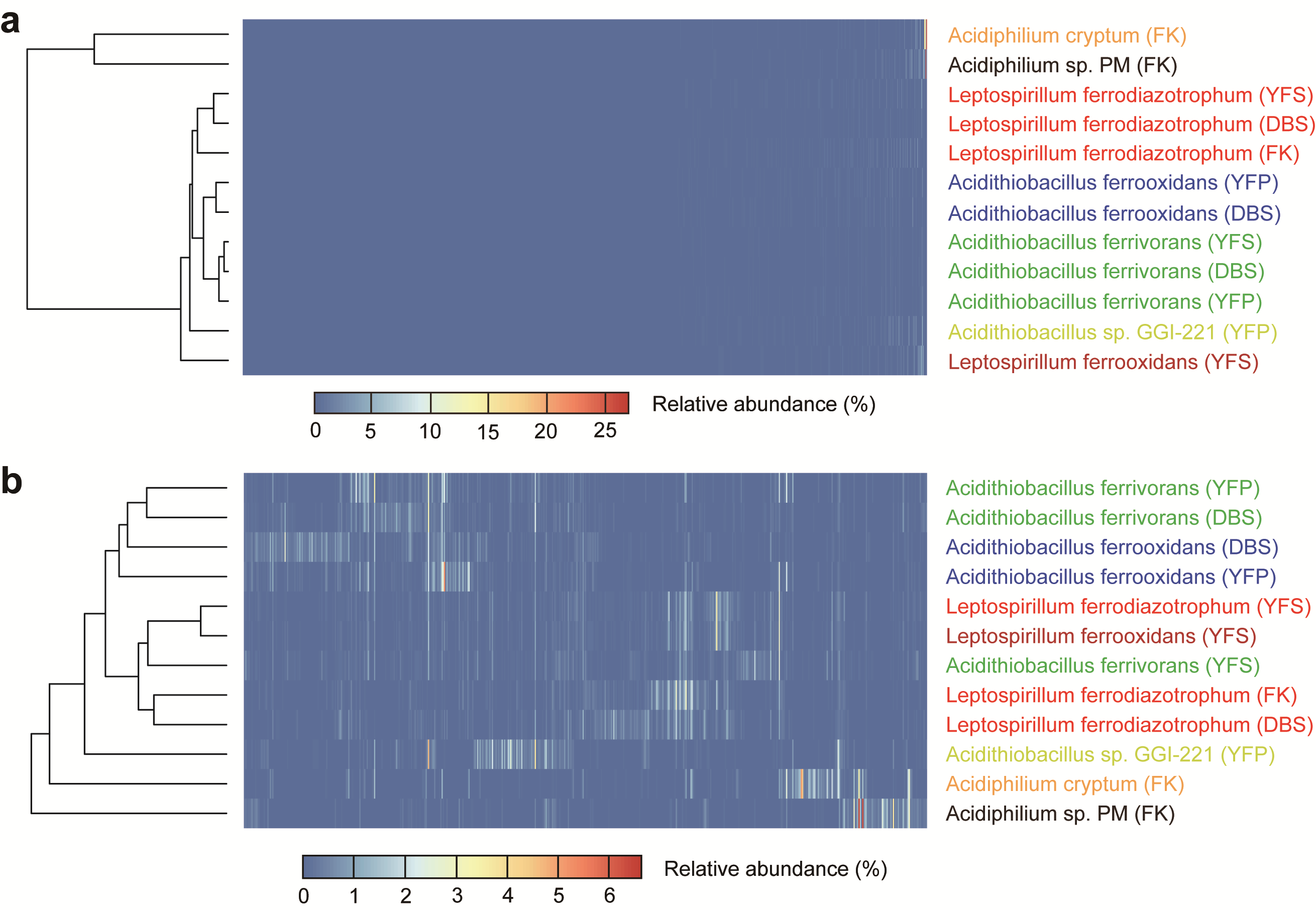


**Figure S5** Hierarchical clustering (performed with correlation distance and average-linkage method using the R package of ‘pheatmap’) of the top 3 most active taxa in the AMD samples based on the relative abundance of (a) DNA COGs and (b) transcript (cDNA) COGs. The relative abundance of each COG represented its proportion in the total transcript pool of each taxon.

**Table S1** Summary of DNA and cDNA pyrosequencing datasets of the four AMD samples

| *Number of* |  | DNA datasets | | | |  | cDNA datasets | | | |
| --- | --- | --- | --- | --- | --- | --- | --- | --- | --- | --- |
|  |  | DBS | FK | YFS | YFP |  | DBS | FK | YFS | YFP |
|  |  |  |  |  |  |  |  |  |  |  |
| Raw sequences |  | 1002956 | 933774 | 762262 | 888497 |  | 252356 | 397736 | 359776 | 282068 |
| rRNA gene sequences ^a^ |  | 4495 (0.4) | 2165 (0.2) | 3931 (0.5) | 3928 (0.4) |  | 117797 (46.7) | 274654 (69.1) | 240437 (66.9) | 167607 (59.4) |
| Replicate sequences ^b^ |  | 65588 (6.6) | 62033 (6.7) | 59531 (7.8) | 58166 (6.6) |  | 14205 (5.6) | 11648 (2.9) | 11063 (3.0) | 14774 (5.3) |
| Non-rRNA, non-replicate sequences |  | 932873 (93.0) | 869576 (93.1) | 698800 (91.7) | 826403 (93.0) |  | 120354 (47.7) | 111434 (28.0) | 108276 (30.1) | 99687 (35.3) |
| NCBI-nr sequences ^c^ |  | 659820 (70.7) | 555628 (63.9) | 564145 (80.7) | 611037 (73.9) |  | 52476 (43.6) | 64502 (58.1) | 60968 (56.3) | 63570 (63.8) |
| Bacteria/Archaea sequences ^d^ |  | 582212 (62.4) | 467560 (53.8) | 524314 (75.0) | 547371 (66.2) |  | 50137 (41.7) | 61816 (55.5) | 58536 (54.1) | 62009 (62.2) |
| COG genes ^e^ |  | 448819 (77.1) | 299152 (64.0) | 437426 (83.4) | 406010 (74.2) |  | 34712 (69.2) | 41107 (66.5) | 38466 (65.7) | 46691 (75.3) |
| KEGG genes ^f^ |  | 522213 (89.7) | 392584 (84.0) | 493494 (94.1) | 486999 (89.0) |  | 42271 (84.3) | 46417 (75.1) | 44227 (75.5) | 46466 (75.0) |
| Assigned to KOs |  | 302914 (52.0) | 189079 (40.4) | 300875 (57.4) | 264937 (48.4) |  | 28363 (56.6) | 30530 (49.4) | 31955 (54.6) | 31880 (51.4) |
| Assigned to KEGG pathways |  | 187075 (32.1) | 114070 (24.4) | 187347 (35.7) | 163473 (29.9) |  | 20506 (40.9) | 20425 (33.0) | 24137 (41.2) | 24518 (39.5) |
|  |  |  |  |  |  |  |  |  |  |  |

Abbreviation: cDNA, complementary DNA; rRNA, ribosomal RNA; NCBI-nr, National Center for Biotechnology Information non-redundant; COG, Clusters of Orthologous Groups of proteins; KEGG, Kyoto Encyclopedia of Genes and Genomes.

^a^ The sequences matched with SSU or LSU rRNA genes sequences using BLASTn (bit score ≥ 50).

^b^ Duplicate sequences (sharing 100% nucleotide identity and length) were identified using cd-hit-454, and excluded from the non-rRNA datasets.

^c^ The non-rRNA, non-replicate DNA and cDNA sequences were compared against the NCBI-nr database using BLASTx (bit score ≥ 40).

^d^ Based on the top BLAST references, the sequences with Bacteria or Archaea NCBI-nr hits were retained for further analysis.

^e^ The non-rRNA DNA and cDNA sequences with NBCI-nr Bacteria/Archaea hits were compared against the extended COG database (STRING) using BLASTx (bit score ≥ 40). All those sequences matching COG genes could also be assigned to COGs and COG categories.

^f^ The non-rRNA DNA and cDNA sequences with NBCI-nr Bacteria/Archaea hits were compared against the KEGG database using BLASTx (bit score ≥ 40).

**Table S2** The top 20 most highly abundant NCBI-nr genes, COGs and KOs in the cDNA datasets of the four AMD communities

*The Table is provided as separate file (Supplementary table 2) for it is too large to integrate*

**Table S3** The detailed information of indicator COGs in the four AMD communities

*The Table is provided as separate file (Supplementary table 3) for it is too large to integrate*

**Table S4** Detailed information of genes with significantly different expression activities in *At. ferrivorans* in the communities of DBS, YFS and YFP

*The Table is provided as separate file (Supplementary table 4) for it is too large to integrate*

**Table S5** Detailed information of genes with significantly different expression activities in *L. ferrodiazotrophum* in the communities of DBS, FK and YFS

*The Table is provided as separate file (Supplementary table 5) for it is too large to integrate*
