# Supplementary material for: Comparative metagenomic and metatranscriptomic analyses of microbial communities in acid mine drainage
Source: ISME J. 2014 Dec 23;9(7):1579–92. doi: 10.1038/ismej.2014.245 (PMC4478699; doi:10.1038/ismej.2014.245)
Supplement: Supplementary Table 2 [file ismej2014245x2.pdf]

Table S3 The top 20 most high abundant NCBI-nr genes, COGs and KOs in the cDNA datasets of the four AMD communities

Rank	NCBI-nr ID	Abundance (%)	NCBI-nr annotation	COG	Abundance (%)	COG annotation	KO	Abundance (%)	KO annotation
	**DBS**			**DBS**			**DBS**		
1	YP_004784314.1	1.18	rusticyanin [*Acidithiobacillus ferrivorans* SS3]	COG1850	2.05	Ribulose 1,5-bisphosphate carboxylase, large subunit	K01601	2.50	rbcL; ribulose bisophosphate carboxylase (EC:4.1.1.39)
2	YP_004784671.1	0.43	ribulose bisphosphate carboxylase large chain [*Acidithiobacillus ferrivorans* SS3]	COG0675	1.43	Transposase and inactivated derivatives	K03076	1.58	preprotein translocase SecY
3	YP_004785120.1	0.41	DNA-directed RNA polymerase subunit beta [*Acidithiobacillus ferrivorans* SS3]	COG0201	1.29	Preprotein translocase subunit SecY	K07496	1.50	transposase
4	YP_003642040.1	0.33	transposase, IS605 OrfB family [*Thiomonas intermedia* K12]	COG0085	1.20	DNA-directed RNA polymerase, beta subunit/140 kD subunit	K03043	1.39	rpoB; DNA-directed RNA polymerase subunit beta (EC:2.7.7.6)
5	YP_004750505.1	0.32	phosphate-selective porin O and P [*Acidithiobacillus caldus* SM-1]	COG0568	0.80	DNA-directed RNA polymerase, sigma subunit (sigma70/sigma32)	K02040	0.93	phosphate ABC transporter, periplasmic phosphate-binding protein
6	YP_004785093.1	0.29	preprotein translocase subunit SecY [*Acidithiobacillus ferrivorans* SS3]	COG0459	0.77	Chaperonin GroEL (HSP60 family)	K03086	0.86	sigma 28
7	YP_004783892.1	0.28	RND family efflux transporter MFP subunit [*Acidithiobacillus ferrivorans* SS3]	COG0226	0.76	ABC-type phosphate transport system, periplasmic component	K02994	0.83	rpsH; 30S ribosomal protein S8
8	YP_004784670.1	0.26	ribulose bisphosphate carboxylase small chain [*Acidithiobacillus ferrivorans* SS3]	COG0086	0.70	DNA-directed RNA polymerase, beta' subunit/160 kD subunit	K01611	0.83	S-adenosylmethionine decarboxylase proenzyme (EC:4.1.1.50)
9	YP_004785228.1	0.25	hypothetical protein Acife_2827 [*Acidithiobacillus ferrivorans* SS3]	COG0096	0.68	Ribosomal protein S8	K04077	0.82	groEL; chaperonin GroEL
10	YP_004783991.1	0.25	phosphate ABC transporter substrate-binding protein [*Acidithiobacillus ferrivorans* SS3]	COG1586	0.68	S-adenosylmethionine decarboxylase	K03046	0.79	DNA-directed RNA polymerase, beta' subunit
11	YP_004785035.1	0.21	hypothetical protein Acife_2631 [*Acidithiobacillus ferrivorans* SS3]	COG0480	0.63	Translation elongation factors (GTPases)	K02355	0.75	elongation factor G
12	YP_004784719.1	0.20	10 kDa chaperonin [*Acidithiobacillus ferrivorans* SS3]	COG0090	0.56	Ribosomal protein L2	K02886	0.68	rpl2p; 50S ribosomal protein L2P
13	YP_004785287.1	0.19	S-adenosylmethionine decarboxylase proenzyme [*Acidithiobacillus ferrivorans* SS3]	COG4451	0.52	Ribulose bisphosphate carboxylase small subunit	K02871	0.65	ribosomal protein L13
14	YP_004782892.1	0.19	phosphoketolase [*Acidithiobacillus ferrivorans* SS3]	COG0102	0.52	Ribosomal protein L13	K01602	0.64	cbbS-1; ribulose bisophosphate carboxylase, small subunit (EC:4.1.1.39)
15	EES53213.1	0.19	DNA-directed RNA polymerase, beta subunit [*Leptospirillum ferrodiazotrophum*]	COG0542	0.52	ATPases with chaperone activity, ATP-binding subunit	K00948	0.62	ribose-phosphate pyrophosphokinase (EC:2.7.6.1)
16	YP_004785485.1	0.18	50S ribosomal protein L13 [*Acidithiobacillus ferrivorans* SS3]	COG0462	0.50	Phosphoribosylpyrophosphate synthetase	K02111	0.61	ATP synthase F1 subunit alpha
17	ZP_09996916.1	0.18	s-adenosylmethionine decarboxylase proenzyme [*Acidithiobacillus thiooxidans* ATCC 19377]	COG0056	0.50	F0F1-type ATP synthase, alpha subunit	K02358	0.54	tuf; elongation factor Tu (EC:3.6.5.3)
18	EIJ76123.1	0.18	hypothetical protein C75L2_00230031 [*Leptospirillum* sp. Group II 'C75']	COG0451	0.50	Nucleoside-diphosphate-sugar epimerases	K00940	0.53	nucleoside-diphosphate kinase (EC:2.7.4.6)
19	YP_004783980.1	0.18	ribosomal RNA large subunit methyltransferase N [*Acidithiobacillus ferrivorans* SS3]	COG0841	0.47	Cation/multidrug efflux pump	K04078	0.50	groS2; chaperonin GroS
20	EES52414.1	0.17	Pyridine nucleotide-disulphide oxidoreductase [*Leptospirillum ferrodiazotrophum*]	COG0050	0.45	GTPases - translation elongation factors	K03798	0.44	ATP-dependent metalloprotease FtsH (EC:3.6.4.6)
	**FK**			**FK**			**FK**		
1	EES54014.1	0.81	Cytochrome c oxidase, subunit I [*Leptospirillum ferrodiazotrophum*]	COG0459	1.80	Chaperonin GroEL (HSP60 family)	K06890	1.83	hypothetical protein
2	YP_004282767.1	0.67	hypothetical protein ACMV_05380 [*Acidiphilium multivorum* AIU301]	COG0477	1.50	Permeases of the major facilitator superfamily	K04077	1.27	groEL; chaperonin GroEL
3	YP_004284525.1	0.55	hypothetical protein ACMV_22960 [*Acidiphilium multivorum* AIU301]	COG0683	1.37	ABC-type branched-chain amino acid transport systems, periplasmic component	K03798	1.24	ATP-dependent metalloprotease FtsH (EC:3.6.4.6)
4	EES53093.1	0.52	putative ATP-dependent Clp protease, ATPase subunit [*Leptospirillum ferrodiazotrophum*]	COG0670	1.36	Integral membrane protein, interacts with FtsH	K03696	1.14	chaperone protein ClpB
5	ZP_08633910.1	0.51	Pyrrolo-quinoline quinone [*Acidiphilium* sp. PM]	COG0542	1.25	ATPases with chaperone activity, ATP-binding subunit	K00404	0.99	cytochrome-c oxidase (EC:1.9.3.1)
6	ZP_08632684.1	0.49	Extracellular ligand-binding receptor [*Acidiphilium* sp. PM]	COG1032	1.10	Fe-S oxidoreductase	K04043	0.96	dnaK; molecular chaperone DnaK
7	YP_001235146.1	0.42	N-acetylmuramoyl-L-alanine amidase [*Acidiphilium* cryptum JF-5]	COG4993	1.03	Glucose dehydrogenase	K01448	0.86	N-acetylmuramoyl-L-alanine amidase (EC:3.5.1.28)
8	EES53529.1	0.41	Peptidase M41, FtsH [*Leptospirillum ferrodiazotrophum*]	COG0465	0.92	ATP-dependent Zn proteases	K00335	0.79	NADH dehydrogenase (quinone) (EC:1.6.99.5)
9	EES53753.1	0.40	AAA ATPase, central domain protein [*Leptospirillum ferrodiazotrophum*]	COG1879	0.89	ABC-type sugar transport system, periplasmic component	K00169	0.74	pyruvate:ferredoxin oxidoreductase, alpha subunit
10	ZP_08635032.1	0.39	hypothetical protein APM_0356 [*Acidiphilium* sp. PM]	COG0443	0.84	Molecular chaperone	K02406	0.72	flagellin domain protein
11	YP_001233562.1	0.36	MarR family transcriptional regulator [*Acidiphilium cryptum* JF-5]	COG1894	0.80	NADH:ubiquinone oxidoreductase, NADH-binding (51 kD) subunit	K01999	0.70	branched-chain amino acid ABC transporter, periplasmic amino acid-binding protein, putative
12	YP_001234015.1	0.35	hypothetical protein Acry_0877 [*Acidiphilium cryptum* JF-5]	COG0531	0.78	Amino acid transporters	K01681	0.63	aconitate hydratase
13	EES53555.1	0.33	cytochrome c, class I [*Leptospirillum ferrodiazotrophum*]	COG0582	0.70	Integrase	K02058	0.58	putative sugar ABC transporter, binding protein
14	EES52967.1	0.29	flagellin domain protein [*Leptospirillum ferrodiazotrophum*]	COG2885	0.66	Outer membrane protein and related peptidoglycan-associated (lipo)proteins	K02274	0.56	cytochrome-c oxidase (EC:1.9.3.1)
15	ZP_08631937.1	0.27	Two component transcriptional regulator [*Acidiphilium* sp. PM]	COG0860	0.64	N-acetylmuramoyl-L-alanine amidase	K01915	0.55	glutamine synthetase
16	EES52770.1	0.27	UDP-N-acetylmuramate--alanine ligase [*Leptospirillum ferrodiazotrophum*]	COG0745	0.64	Response regulators consisting of a CheY-like receiver domain and a winged-helix DNA-binding domain	K01338	0.55	K01338 ATP-dependent Lon protease [EC:3.4.21.53]
17	EES52036.1	0.27	Radical SAM domain protein [*Leptospirillum ferrodiazotrophum*]	COG1846	0.60	Transcriptional regulators	K03544	0.51	clpX; ATP-dependent Clp protease ATP-binding subunit ClpX
18	EES53715.1	0.26	NADH dehydrogenase (quinone) F subunit [*Leptospirillum ferrodiazotrophum*]	COG0464	0.59	ATPases of the AAA+ class	K03076	0.50	preprotein translocase SecY
19	EES52069.1	0.25	putative cobalamin B12-binding/Radical SAM family protein [*Leptospirillum ferrodiazotrophum*]	COG1344	0.56	Flagellin and related hook-associated proteins	K00170	0.48	thiamine pyrophosphate binding domain-containing protein
20	EES53319.1	0.24	Pyruvate:ferredoxin oxidoreductase alpha subunit [*Leptospirillum ferrodiazotrophum*]	COG1013	0.53	Pyruvate:ferredoxin oxidoreductase and related 2-oxoacid:ferredoxin oxidoreductases, beta subunit	K02355	0.47	elongation factor G
	**YFS**			**YFS**			**YFS**		
1	YP_005468611.1	2.83	cytochrome c oxidase cbb3 type, subunit I [*Leptospirillum ferrooxidans* C2-3]	COG3278	3.03	Cbb3-type cytochrome oxidase, subunit 1	K00404	4.78	cytochrome-c oxidase (EC:1.9.3.1)
2	YP_005468512.1	1.04	cytochrome c oxidase monoheme subunit [*Leptospirillum ferrooxidans* C2-3]	COG0459	2.90	Chaperonin GroEL (HSP60 family)	K04077	3.29	groEL; chaperonin GroEL
3	YP_005469840.1	0.95	cytochrome 579 [*Leptospirillum ferrooxidans* C2-3]	COG0542	1.52	ATPases with chaperone activity, ATP-binding subunit	K01601	1.73	rbcL; ribulose bisophosphate carboxylase (EC:4.1.1.39)
4	YP_005469616.1	0.68	succinyl-CoA synthetase, alpha subunit [*Leptospirillum ferrooxidans* C2-3]	COG1850	1.46	Ribulose 1,5-bisphosphate carboxylase, large subunit	K04043	1.52	dnaK; molecular chaperone DnaK
5	YP_005470141.1	0.61	pyruvate synthase, alpha subunit [*Leptospirillum ferrooxidans* C2-3]	COG0443	1.35	Molecular chaperone	K00169	1.45	pyruvate:ferredoxin oxidoreductase, alpha subunit
6	YP_005469045.1	0.60	ATP-dependent Clp protease, ATPase subunit [*Leptospirillum ferrooxidans* C2-3]	COG0674	1.16	Pyruvate:ferredoxin oxidoreductase and related 2-oxoacid:ferredoxin oxidoreductases, alpha subunit	K03696	1.25	chaperone protein ClpB
7	YP_005470139.1	0.58	ferredoxin oxidoreductase, gamma subunit [*Leptospirillum ferrooxidans* C2-3]	COG1014	1.02	Pyruvate:ferredoxin oxidoreductase and related 2-oxoacid:ferredoxin oxidoreductases, gamma subunit	K00172	1.23	pyruvate/ketoisovalerate oxidoreductase, gamma subunit
8	YP_005469357.1	0.58	hypothetical protein LFE_1545 [*Leptospirillum ferrooxidans* C2-3]	COG0074	1.00	Succinyl-CoA synthetase, alpha subunit	K02406	1.15	flagellin domain protein
9	YP_005469896.1	0.47	fliC gene product [*Leptospirillum ferrooxidans* C2-3]	COG1344	1.00	Flagellin and related hook-associated proteins	K03043	1.14	rpoB; DNA-directed RNA polymerase subunit beta (EC:2.7.7.6)
10	YP_005469282.1	0.41	dnaK gene product [*Leptospirillum ferrooxidans* C2-3]	COG0085	0.96	DNA-directed RNA polymerase, beta subunit/140 kD subunit	K02994	1.12	rpsH; 30S ribosomal protein S8
11	YP_005469567.1	0.37	4-hydroxy-3-methylbut-2-enyl-diphosphate reductase [*Leptospirillum ferrooxidans* C2-3]	COG0096	0.93	Ribosomal protein S8	K01915	1.10	glutamine synthetase
12	YP_005468614.1	0.36	isocitrate dehydrogenase [*Leptospirillum ferrooxidans* C2-3]	COG0174	0.90	Glutamine synthetase	K15233	1.08	sucD1; succinyl-CoA ligase alpha subunit
13	YP_005469655.1	0.34	cysteine desulfurase [*Leptospirillum ferrooxidans* C2-3]	COG0473	0.84	Isocitrate/isopropylmalate dehydrogenase	K04487	0.82	aminotransferase class V
14	YP_005469614.1	0.34	aconitate hydratase [*Leptospirillum ferrooxidans* C2-3]	COG2993	0.72	Cbb3-type cytochrome oxidase, cytochrome c subunit	K02355	0.79	elongation factor G
15	YP_005469816.1	0.31	hypothetical protein LFE_2011 [*Leptospirillum ferrooxidans* C2-3]	COG1104	0.69	Cysteine sulfinate desulfinase/cysteine desulfurase and related enzymes	K01681	0.79	aconitate hydratase
16	YP_005469448.1	0.28	thiamine biosynthesis protein [*Leptospirillum ferrooxidans* C2-3]	COG1032	0.68	Fe-S oxidoreductase	K02111	0.75	ATP synthase F1 subunit alpha
17	YP_005470138.1	0.28	pyruvate ferredoxin oxidoreductase, epsiron subunit [*Leptospirillum ferrooxidans* C2-3]	COG0480	0.68	Translation elongation factors (GTPases)	K00030	0.73	isocitrate dehydrogenase, NAD-dependent (EC:1.1.1.41)
18	YP_005469331.1	0.27	chaperonin GroEL [*Leptospirillum ferrooxidans* C2-3]	COG1048	0.65	Aconitase A	K03798	0.72	ATP-dependent metalloprotease FtsH (EC:3.6.4.6)
19	YP_005469566.1	0.23	radical SAM family protein [*Leptospirillum ferrooxidans* C2-3]	COG0056	0.63	F0F1-type ATP synthase, alpha subunit	K01602	0.72	cbbS-1; ribulose bisophosphate carboxylase, small subunit (EC:4.1.1.39)
20	YP_005468709.1	0.23	DNA-directed RNA polymerase subunit beta [*Leptospirillum ferrooxidans* C2-3]	COG4451	0.60	Ribulose bisphosphate carboxylase small subunit	K02040	0.68	phosphate ABC transporter, periplasmic phosphate-binding protein
	**YFP**			**YFP**			**YFP**		
1	YP_004784314.1	2.22	rusticyanin [*Acidithiobacillus ferrivorans* SS3]	COG0459	2.93	Chaperonin GroEL (HSP60 family)	K04077	3.64	groEL; chaperonin GroEL
2	YP_004783991.1	0.92	phosphate ABC transporter substrate-binding protein [*Acidithiobacillus ferrivorans* SS3]	COG1850	2.71	Ribulose 1,5-bisphosphate carboxylase, large subunit	K01601	3.41	rbcL; ribulose bisophosphate carboxylase (EC:4.1.1.39)
3	YP_004783142.1	0.62	cytochrome b/b6 domain-containing protein [*Acidithiobacillus ferrivorans* SS3]	COG0226	1.96	ABC-type phosphate transport system, periplasmic component	K02040	2.49	phosphate ABC transporter, periplasmic phosphate-binding protein
4	YP_004782650.1	0.62	cytochrome c-type biogenesis protein CcsB [*Acidithiobacillus ferrivorans* SS3]	COG4451	1.80	Ribulose bisphosphate carboxylase small subunit	K01602	2.25	cbbS-1; ribulose bisophosphate carboxylase, small subunit (EC:4.1.1.39)
5	YP_004784318.1	0.60	cytochrome c oxidase subunit I [*Acidithiobacillus ferrivorans* SS3]	COG1290	1.53	Cytochrome b subunit of the bc complex	K02274	1.92	cytochrome-c oxidase (EC:1.9.3.1)
6	YP_004784720.1	0.55	60 kDa chaperonin [*Acidithiobacillus ferrivorans* SS3]	COG0843	1.53	Heme/copper-type cytochrome/quinol oxidases, subunit 1	K03043	1.76	rpoB; DNA-directed RNA polymerase subunit beta (EC:2.7.7.6)
7	YP_004784322.1	0.50	cytochrome c [*Acidithiobacillus ferrivorans* SS3]	COG0085	1.50	DNA-directed RNA polymerase, beta subunit/140 kD subunit	K00412	1.73	qcrB; quinol-cytochrome c reductase, fused cytochrome b/c subunit (EC:1.10.2.2)
8	YP_004785093.1	0.49	preprotein translocase subunit SecY [*Acidithiobacillus ferrivorans* SS3]	COG0201	1.33	Preprotein translocase subunit SecY	K03076	1.61	preprotein translocase SecY
9	YP_003525267.1	0.48	cytochrome C oxidase [*Sideroxydans lithotrophicus* ES-1]	COG0326	1.22	Molecular chaperone, HSP90 family	K04079	1.56	htpG; chaperone HSP90
10	YP_002221107.1	0.47	cytochrome b/b6 domain-containing protein [*Acidithiobacillus ferrooxidans* ATCC 53993]	COG0755	1.22	ABC-type transport system involved in cytochrome c biogenesis, permease component	K02358	1.42	tuf; elongation factor Tu (EC:3.6.5.3)
11	YP_004785120.1	0.46	DNA-directed RNA polymerase subunit beta [*Acidithiobacillus ferrivorans* SS3]	COG0050	1.19	GTPases - translation elongation factors	K02275	1.31	cytochrome c oxidase subunit II
12	YP_004782640.1	0.44	outer membrane porin [*Acidithiobacillus ferrivorans* SS3]	COG2863	1.15	Cytochrome c553	K02946	1.06	rps10p; 30S ribosomal protein S10P
13	YP_004783144.1	0.44	short-chain dehydrogenase/reductase SDR [*Acidithiobacillus ferrivorans* SS3]	COG1622	1.06	Heme/copper-type cytochrome/quinol oxidases, subunit 2	K01338	1.03	K01338 ATP-dependent Lon protease [EC:3.4.21.53]
14	YP_004784671.1	0.40	ribulose bisphosphate carboxylase large chain [*Acidithiobacillus ferrivorans* SS3]	COG1028	0.91	Dehydrogenases with different specificities (related to short-chain alcohol dehydrogenases)	K04043	0.97	dnaK; molecular chaperone DnaK
15	YP_004784670.1	0.37	ribulose bisphosphate carboxylase small chain [*Acidithiobacillus ferrivorans* SS3]	COG0051	0.85	Ribosomal protein S10	K02864	0.94	rplP0; acidic ribosomal protein P0
16	YP_004785113.1	0.36	50S ribosomal protein L3 [*Acidithiobacillus ferrivorans* SS3]	COG0443	0.84	Molecular chaperone	K00404	0.89	cytochrome-c oxidase (EC:1.9.3.1)
17	YP_004784719.1	0.35	10 kDa chaperonin [*Acidithiobacillus ferrivorans* SS3]	COG0568	0.83	DNA-directed RNA polymerase, sigma subunit (sigma70/sigma32)	K02111	0.87	ATP synthase F1 subunit alpha
18	ZP_09996542.1	0.35	30S ribosomal protein S10 [*Acidithiobacillus thiooxidans* ATCC 19377]	COG0466	0.81	ATP-dependent Lon protease, bacterial type	K02355	0.86	elongation factor G
19	YP_004785539.1	0.34	ribulose bisphosphate carboxylase large chain [*Acidithiobacillus ferrivorans* SS3]	COG0244	0.79	Ribosomal protein L10	K02906	0.84	rpl3p; 50S ribosomal protein L3P
20	YP_004784319.1	0.34	cytochrome c oxidase subunit II [*Acidithiobacillus ferrivorans* SS3]	COG0542	0.79	ATPases with chaperone activity, ATP-binding subunit	K02886	0.71	rpl2p; 50S ribosomal protein L2P
