# Supplementary material for: Comparative metagenomic and metatranscriptomic analyses of microbial communities in acid mine drainage
Source: ISME J. 2014 Dec 23;9(7):1579–92. doi: 10.1038/ismej.2014.245 (PMC4478699; doi:10.1038/ismej.2014.245)
Supplement: Supplementary Table 3 [file ismej2014245x3.pdf]

Table S3 The detailed information of indicator COGs in the four AMD communities

COG	DBS-cDNA	FK-cDNA	YFS-cDNA	YFP-cDNA	COG category	COG annotation
**DBS**						
COG0355	**0.2045%**	0.0389%	0.0780%	0.0814%	C	F0F1-type ATP synthase, epsilon subunit (mitochondrial delta subunit)
COG0356	**0.3083%**	0.1727%	0.1794%	0.0835%	C	F0F1-type ATP synthase, subunit a
COG0426	**0.1008%**	0.0389%	0.0234%	0.0450%	C	Uncharacterized flavoproteins
COG0757	**0.0605%**	0.0049%	0.0026%	0.0214%	E	3-dehydroquinate dehydratase II
COG1605	**0.1152%**	0.0243%	0.0338%	0.0193%	E	Chorismate mutase
COG2856	**0.0979%**	0.0049%	0.0156%	0.0214%	E	Predicted Zn peptidase
COG1586	**0.6770%**	0.1265%	0.2028%	0.1885%	E	S-adenosylmethionine decarboxylase
COG0175	**0.1786%**	0.0414%	0.0728%	0.0750%	EH	3'-phosphoadenosine 5'-phosphosulfate sulfotransferase (PAPS reductase)/FAD synthetase and related enzymes
COG0512	**0.0691%**	0.0146%	0.0260%	0.0171%	EH	Anthranilate/para-aminobenzoate synthases component II
COG0504	**0.2478%**	0.0316%	0.1300%	0.1028%	F	CTP synthase (UTP-ammonia lyase)
COG0105	**0.4292%**	0.0462%	0.0286%	0.0386%	F	Nucleoside diphosphate kinase
COG0462	**0.5041%**	0.1022%	0.0962%	0.0514%	FE	Phosphoribosylpyrophosphate synthetase
COG0483	**0.0893%**	0.0268%	0.0234%	0.0086%	G	Archaeal fructose-1,6-bisphosphatase and related enzymes of inositol monophosphatase family
COG3957	**0.3572%**	0.0268%	0.1508%	0.2313%	G	Phosphoketolase
COG2227	**0.1066%**	0.0024%	0.0078%	0.0086%	H	2-polyprenyl-3-methyl-5-hydroxy-6-metoxy-1,4-benzoquinol methylase
COG1985	**0.0519%**	0.0146%	0.0052%	0.0043%	H	Pyrimidine reductase, riboflavin biosynthesis
COG2104	**0.0490%**	0.0097%	0.0026%	0.0064%	H	Sulfur transfer protein involved in thiamine biosynthesis
COG1154	**0.1988%**	0.1192%	0.0910%	0.0942%	HI	Deoxyxylulose-5-phosphate synthase
COG1947	**0.0864%**	0.0073%	0.0104%	0.0193%	I	4-diphosphocytidyl-2C-methyl-D-erythritol 2-phosphate synthase
COG0511	**0.1066%**	0.0122%	0.0156%	0.0086%	I	Biotin carboxyl carrier protein
COG1398	**0.1757%**	0.0000%	0.0936%	0.0171%	I	Fatty-acid desaturase
COG1502	**0.0691%**	0.0122%	0.0104%	0.0193%	I	Phosphatidylserine/phosphatidylglycerophosphate/cardiolipin synthases and related enzymes
COG0008	**0.1786%**	0.0997%	0.0754%	0.0750%	J	Glutamyl- and glutaminyl-tRNA synthetases
COG1190	**0.1037%**	0.0170%	0.0364%	0.0300%	J	Lysyl-tRNA synthetase (class II)
COG1530	**0.1613%**	0.0632%	0.0572%	0.0835%	J	Ribonucleases G and E

COG	DBS-cDNA	FK-cDNA	YFS-cDNA	YFP-cDNA	COG category	COG annotation
**DBS**						
COG0102	**0.5243%**	0.1630%	0.1768%	0.2656%	J	Ribosomal protein L13
COG0335	**0.1700%**	0.0462%	0.0286%	0.0814%	J	Ribosomal protein L19
COG1825	**0.1527%**	0.0560%	0.0520%	0.0214%	J	Ribosomal protein L25 (general stress protein Ctc)
COG0227	**0.1584%**	0.0535%	0.0520%	0.0300%	J	Ribosomal protein L28
COG0267	**0.2708%**	0.0268%	0.1014%	0.0321%	J	Ribosomal protein L33
COG0359	**0.1642%**	0.0511%	0.0520%	0.0578%	J	Ribosomal protein L9
COG0048	**0.1412%**	0.0292%	0.0624%	0.0728%	J	Ribosomal protein S12
COG0268	**0.0893%**	0.0268%	0.0208%	0.0257%	J	Ribosomal protein S20
COG0828	**0.1671%**	0.0389%	0.0468%	0.0064%	J	Ribosomal protein S21
COG0360	**0.1901%**	0.0365%	0.0728%	0.1135%	J	Ribosomal protein S6
COG0858	**0.0461%**	0.0073%	0.0078%	0.0021%	J	Ribosome-binding factor A
COG0594	**0.0375%**	0.0000%	0.0000%	0.0064%	J	RNase P protein component
COG0231	**0.1642%**	0.0316%	0.0390%	0.0857%	J	Translation elongation factor P (EF-P)/translation initiation factor 5A (eIF-5A)
COG1508	**0.0835%**	0.0341%	0.0234%	0.0321%	K	DNA-directed RNA polymerase specialized sigma subunit, sigma54 homolog
COG0571	**0.1210%**	0.0341%	0.0312%	0.0643%	K	dsRNA-specific ribonuclease
COG0553	**0.1757%**	0.0754%	0.0676%	0.0493%	KL	Superfamily II DNA/RNA helicases, SNF2 family
COG3392	**0.0519%**	0.0024%	0.0078%	0.0021%	L	Adenine-specific DNA methylase
COG0323	**0.0691%**	0.0073%	0.0078%	0.0150%	L	DNA mismatch repair enzyme (predicted ATPase)
COG0305	**0.2161%**	0.0268%	0.0728%	0.1157%	L	Replicative DNA helicase
COG3344	**0.1383%**	0.0584%	0.0702%	0.0750%	L	Retron-type reverse transcriptase
COG0338	**0.0720%**	0.0146%	0.0208%	0.0150%	L	Site-specific DNA methylase
COG0270	**0.1124%**	0.0097%	0.0416%	0.0257%	L	Site-specific DNA methylase
COG0210	**0.2794%**	0.0560%	0.0832%	0.0814%	L	Superfamily I DNA and RNA helicases
COG0550	**0.0576%**	0.0049%	0.0182%	0.0150%	L	Topoisomerase IA
COG3436	**0.1095%**	0.0365%	0.0260%	0.0386%	L	Transposase and inactivated derivatives
COG3464	**0.1873%**	0.0608%	0.0520%	0.0257%	L	Transposase and inactivated derivatives

COG	DBS-cDNA	FK-cDNA	YFS-cDNA	YFP-cDNA	COG category	COG annotation
**DBS**						
COG3328	**0.2852%**	0.0608%	0.0208%	0.0343%	L	Transposase and inactivated derivatives
COG0675	**1.4347%**	0.3333%	0.2938%	0.1542%	L	Transposase and inactivated derivatives
COG1197	**0.0634%**	0.0170%	0.0156%	0.0086%	LK	Transcription-repair coupling factor (superfamily II helicase)
COG0513	**0.2996%**	0.0341%	0.1014%	0.1221%	LKJ	Superfamily II DNA and RNA helicases
COG1088	**0.0749%**	0.0243%	0.0130%	0.0150%	M	dTDP-D-glucose 4,6-dehydratase
COG0463	**0.3227%**	0.1533%	0.2184%	0.2035%	M	Glycosyltransferases involved in cell wall biogenesis
COG0739	**0.1671%**	0.0389%	0.0598%	0.0129%	M	Membrane proteins related to metalloendopeptidases
COG0845	**0.4091%**	0.0900%	0.1508%	0.2013%	M	Membrane-fusion protein
COG2089	**0.0519%**	0.0049%	0.0130%	0.0107%	M	Sialic acid synthase
COG0774	**0.1613%**	0.0657%	0.0702%	0.0600%	M	UDP-3-O-acyl-N-acetylglucosamine deacetylase
COG1352	**0.0778%**	0.0316%	0.0208%	0.0021%	NT	Methylase of chemotaxis methyl-accepting proteins
COG3167	**0.0835%**	0.0049%	0.0052%	0.0150%	NU	Tfp pilus assembly protein PilO
COG4967	**0.0288%**	0.0000%	0.0000%	0.0000%	NU	Tfp pilus assembly protein PilV
COG0760	**0.2593%**	0.1095%	0.1638%	0.1692%	O	Parvulin-like peptidyl-prolyl isomerase
COG2377	**0.1095%**	0.0122%	0.0364%	0.0236%	O	Predicted molecular chaperone distantly related to HSP70-fold metalloproteases
COG0803	**0.0547%**	0.0122%	0.0052%	0.0129%	P	ABC-type metal ion transport system, periplasmic component/surface adhesin
COG3221	**0.0778%**	0.0024%	0.0078%	0.0193%	P	ABC-type phosphate/phosphonate transport system, periplasmic component
COG1230	**0.0835%**	0.0122%	0.0130%	0.0257%	P	Co/Zn/Cd efflux system component
COG0704	**0.1210%**	0.0268%	0.0494%	0.0514%	P	Phosphate uptake regulator
COG3696	**0.2852%**	0.0438%	0.0650%	0.0685%	P	Putative silver efflux pump
COG0500	**0.2276%**	0.1289%	0.1300%	0.1199%	QR	SAM-dependent methyltransferases
COG0784	**0.1815%**	0.0487%	0.0858%	0.0428%	T	FOG: CheY-like receiver
COG1217	**0.2622%**	0.1654%	0.1690%	0.0857%	T	Predicted membrane GTPase involved in stress response
COG2197	**0.1124%**	0.0122%	0.0312%	0.0043%	TK	Response regulator containing a CheY-like receiver domain and an HTH DNA-binding domain
COG4965	**0.0634%**	0.0000%	0.0000%	0.0021%	U	Flp pilus assembly protein TadB
COG4964	**0.0432%**	0.0073%	0.0000%	0.0086%	U	Flp pilus assembly protein, secretin CpaC

COG	DBS-cDNA	FK-cDNA	YFS-cDNA	YFP-cDNA	COG category	COG annotation
**DBS**						
COG0706	**0.1354%**	0.0535%	0.0702%	0.0664%	U	Preprotein translocase subunit YidC
COG2274	**0.0605%**	0.0049%	0.0104%	0.0000%	V	ABC-type bacteriocin/lantibiotic exporters, contain an N-terminal double-glycine peptidase domain
COG2367	**0.0317%**	0.0000%	0.0000%	0.0000%	V	Beta-lactamase class A
COG0841	**0.4725%**	0.3381%	0.3250%	0.2870%	V	Cation/multidrug efflux pump
COG0286	**0.3918%**	0.0560%	0.0442%	0.0535%	V	Type I restriction-modification system methyltransferase subunit
COG0610	**0.1469%**	0.0511%	0.0364%	0.0514%	V	Type I site-specific restriction-modification system, R (restriction) subunit and related helicases
COG0843	**0.0749%**	0.5133%	0.2028%	1.5292%	C	Heme/copper-type cytochrome/quinol oxidases, subunit 1
COG1012	**0.0490%**	0.2603%	0.1066%	0.1435%	C	NAD-dependent aldehyde dehydrogenases
COG0542	**0.5214%**	1.2504%	1.5208%	0.7903%	O	ATPases with chaperone activity, ATP-binding subunit
COG1219	**0.2074%**	0.3771%	0.3432%	0.3363%	O	ATP-dependent protease Clp, ATPase subunit
COG3278	**0.1815%**	0.4817%	3.0338%	0.3684%	O	Cbb3-type cytochrome oxidase, subunit 1
COG0459	**0.7749%**	1.8002%	2.8961%	2.9342%	O	Chaperonin GroEL (HSP60 family)
COG0443	**0.3918%**	0.8393%	1.3492%	0.8396%	O	Molecular chaperone
**FK**						
COG1529	0.0086%	**0.2725%**	0.0156%	0.0364%	C	Aerobic-type carbon monoxide dehydrogenase, large subunit CoxL/CutL homologs
COG1319	0.0000%	**0.1825%**	0.0000%	0.0171%	C	Aerobic-type carbon monoxide dehydrogenase, middle subunit CoxM/CutM homologs
COG2080	0.0086%	**0.1046%**	0.0156%	0.0086%	C	Aerobic-type carbon monoxide dehydrogenase, small subunit CoxS/CutS homologs
COG1454	0.0000%	**0.2992%**	0.0078%	0.0086%	C	Alcohol dehydrogenase, class IV
COG1156	0.0000%	**0.0535%**	0.0026%	0.0064%	C	Archaeal/vacuolar-type H+-ATPase subunit B
COG1271	0.0029%	**0.0730%**	0.0286%	0.0171%	C	Cytochrome bd-type quinol oxidase, subunit 1
COG3474	0.0029%	**0.2116%**	0.0052%	0.0171%	C	Cytochrome c2
COG4654	0.0144%	**0.1752%**	0.0234%	0.1049%	C	Cytochrome c551/c552
COG2025	0.0144%	**0.0560%**	0.0156%	0.0150%	C	Electron transfer flavoprotein, alpha subunit
COG2086	0.0144%	**0.0608%**	0.0208%	0.0086%	C	Electron transfer flavoprotein, beta subunit
COG1032	0.3371%	**1.1044%**	0.6811%	0.2442%	C	Fe-S oxidoreductase
COG1143	0.0691%	**0.1387%**	0.0546%	0.0214%	C	Formate hydrogenlyase subunit 6/NADH:ubiquinone oxidoreductase 23 kD subunit (chain I)

COG	DBS-cDNA	FK-cDNA	YFS-cDNA	YFP-cDNA	COG category	COG annotation
**FK**						
COG2224	0.0000%	**0.2676%**	0.0052%	0.0043%	C	Isocitrate lyase
COG1282	0.0000%	**0.0754%**	0.0234%	0.0129%	C	NAD/NADP transhydrogenase beta subunit
COG1012	0.0490%	**0.2603%**	0.1066%	0.1435%	C	NAD-dependent aldehyde dehydrogenases
COG1902	0.0490%	**0.2092%**	0.0754%	0.0321%	C	NADH:flavin oxidoreductases, Old Yellow Enzyme family
COG3761	0.0000%	**0.0195%**	0.0000%	0.0000%	C	NADH:ubiquinone oxidoreductase 17.2 kD subunit
COG1905	0.0519%	**0.1168%**	0.0416%	0.0493%	C	NADH:ubiquinone oxidoreductase 24 kD subunit
COG0839	0.0288%	**0.0876%**	0.0364%	0.0214%	C	NADH:ubiquinone oxidoreductase subunit 6 (chain J)
COG1894	0.3227%	**0.7979%**	0.5537%	0.0900%	C	NADH:ubiquinone oxidoreductase, NADH-binding (51 kD) subunit
COG2009	0.0000%	**0.0341%**	0.0000%	0.0086%	C	Succinate dehydrogenase/fumarate reductase, cytochrome b subunit
COG1139	0.0000%	**0.0462%**	0.0104%	0.0021%	C	Uncharacterized conserved protein containing a ferredoxin-like domain
COG0604	0.0000%	**0.1241%**	0.0234%	0.0043%	CR	NADPH:quinone reductase and related Zn-dependent oxidoreductases
COG0772	0.0202%	**0.0681%**	0.0104%	0.0214%	D	Bacterial cell division membrane protein
COG0160	0.0029%	**0.1216%**	0.0078%	0.0043%	E	4-aminobutyrate aminotransferase and related aminotransferases
COG4177	0.0029%	**0.0632%**	0.0052%	0.0236%	E	ABC-type branched-chain amino acid transport system, permease component
COG0411	0.0058%	**0.0754%**	0.0000%	0.0064%	E	ABC-type branched-chain amino acid transport systems, ATPase component
COG0683	0.0403%	**1.3672%**	0.0494%	0.1114%	E	ABC-type branched-chain amino acid transport systems, periplasmic component
COG4608	0.0058%	**0.0681%**	0.0234%	0.0064%	E	ABC-type oligopeptide transport system, ATPase component
COG1126	0.0000%	**0.0219%**	0.0000%	0.0000%	E	ABC-type polar amino acid transport system, ATPase component
COG1177	0.0000%	**0.0219%**	0.0000%	0.0000%	E	ABC-type spermidine/putrescine transport system, permease component II
COG0531	0.1527%	**0.7833%**	0.1196%	0.1585%	E	Amino acid transporters
COG1812	0.0029%	**0.0560%**	0.0130%	0.0064%	E	Archaeal S-adenosylmethionine synthetase
COG2235	0.0000%	**0.0195%**	0.0000%	0.0000%	E	Arginine deiminase
COG0559	0.0058%	**0.1192%**	0.0156%	0.0214%	E	Branched-chain amino acid ABC-type transport system, permease components
COG0334	0.0086%	**0.0754%**	0.0286%	0.0064%	E	Glutamate dehydrogenase/leucine dehydrogenase
COG0687	0.0000%	**0.1070%**	0.0000%	0.0086%	E	Spermidine/putrescine-binding periplasmic protein
COG0834	0.0000%	**0.0365%**	0.0052%	0.0021%	ET	ABC-type amino acid transport/signal transduction systems, periplasmic component/domain

COG	DBS-cDNA	FK-cDNA	YFS-cDNA	YFP-cDNA	COG category	COG annotation
**FK**						
COG0737	0.0115%	**0.0730%**	0.0208%	0.0193%	F	5'-nucleotidase/2',3'-cyclic phosphodiesterase and related esterases
COG2019	0.0000%	**0.0268%**	0.0000%	0.0043%	F	Archaeal adenylate kinase
COG1129	0.0115%	**0.1508%**	0.0000%	0.0064%	G	ABC-type sugar transport system, ATPase component
COG1653	0.0173%	**0.3649%**	0.2262%	0.0278%	G	ABC-type sugar transport system, periplasmic component
COG1879	0.0173%	**0.8904%**	0.0078%	0.0471%	G	ABC-type sugar transport system, periplasmic component
COG1175	0.0058%	**0.0584%**	0.0156%	0.0129%	G	ABC-type sugar transport systems, permease components
COG4213	0.0000%	**0.0219%**	0.0000%	0.0000%	G	ABC-type xylose transport system, periplasmic component
COG4421	0.0000%	**0.0268%**	0.0000%	0.0000%	G	Capsular polysaccharide biosynthesis protein
COG5309	0.0000%	**0.0195%**	0.0000%	0.0000%	G	Exo-beta-1,3-glucanase
COG4993	0.0144%	**1.0315%**	0.0052%	0.0643%	G	Glucose dehydrogenase
COG1172	0.0029%	**0.2603%**	0.0026%	0.0086%	G	Ribose/xylose/arabinose/galactoside ABC-type transport systems, permease components
COG0235	0.0173%	**0.2603%**	0.0052%	0.0107%	G	Ribulose-5-phosphate 4-epimerase and related epimerases and aldolases
COG1523	0.0115%	**0.0511%**	0.0052%	0.0064%	G	Type II secretory pathway, pullulanase PulA and related glycosidases
COG0477	0.4465%	**1.5010%**	0.3874%	0.4947%	GEPR	Permeases of the major facilitator superfamily
COG1682	0.0230%	**0.1873%**	0.0442%	0.0578%	GM	ABC-type polysaccharide/polyol phosphate export systems, permease component
COG1575	0.0029%	**0.0341%**	0.0000%	0.0000%	H	1,4-dihydroxy-2-naphthoate octaprenyltransferase
COG0720	0.0086%	**0.0438%**	0.0026%	0.0107%	H	6-pyruvoyl-tetrahydropterin synthase
COG1903	0.0173%	**0.0754%**	0.0130%	0.0150%	H	Cobalamin biosynthesis protein CbiD
COG0684	0.0000%	**0.0316%**	0.0000%	0.0000%	H	Demethylmenaquinone methyltransferase
COG1893	0.0000%	**0.0341%**	0.0052%	0.0000%	H	Ketopantoate reductase
COG0320	0.0432%	**0.2238%**	0.0208%	0.0257%	H	Lipoate synthase
COG0214	0.0029%	**0.0316%**	0.0026%	0.0000%	H	Pyridoxine biosynthesis enzyme
COG5424	0.0000%	**0.0389%**	0.0000%	0.0021%	H	Pyrroloquinoline quinone (Coenzyme PQQ) biosynthesis protein C
COG2022	0.0691%	**0.1703%**	0.0572%	0.0985%	H	Uncharacterized enzyme of thiazole biosynthesis
COG1250	0.0605%	**0.1752%**	0.0624%	0.0300%	I	3-hydroxyacyl-CoA dehydrogenase
COG0183	0.0576%	**0.1241%**	0.0416%	0.0214%	I	Acetyl-CoA acetyltransferase

COG	DBS-cDNA	FK-cDNA	YFS-cDNA	YFP-cDNA	COG category	COG annotation
**FK**						
COG2030	0.0115%	**0.0487%**	0.0026%	0.0000%	I	Acyl dehydratase
COG1960	0.0432%	**0.4330%**	0.0416%	0.0428%	I	Acyl-CoA dehydrogenases
COG0657	0.0029%	**0.0705%**	0.0052%	0.0064%	I	Esterase/lipase
COG1884	0.0029%	**0.0632%**	0.0052%	0.0043%	I	Methylmalonyl-CoA mutase, N-terminal domain/subunit
COG4553	0.0000%	**0.0365%**	0.0000%	0.0000%	I	Poly-beta-hydroxyalkanoate depolymerase
COG0318	0.0403%	**0.1752%**	0.0364%	0.0428%	IQ	Acyl-CoA synthetases (AMP-forming)/AMP-acid ligases II
COG3240	0.0000%	**0.0292%**	0.0000%	0.0000%	IR	Phospholipase/lecithinase/hemolysin
COG1384	0.0086%	**0.0535%**	0.0104%	0.0043%	J	Lysyl-tRNA synthetase (class I)
COG1491	0.0000%	**0.0365%**	0.0078%	0.0021%	J	Predicted RNA-binding protein
COG1189	0.0058%	**0.0681%**	0.0234%	0.0000%	J	Predicted rRNA methylase
COG1632	0.0000%	**0.0243%**	0.0000%	0.0000%	J	Ribosomal protein L15E
COG2238	0.0000%	**0.0389%**	0.0000%	0.0021%	J	Ribosomal protein S19E (S16A)
COG1544	0.0979%	**0.2627%**	0.0936%	0.0493%	J	Ribosome-associated protein Y (PSrp-1)
COG2123	0.0029%	**0.0365%**	0.0026%	0.0043%	J	RNase PH-related exoribonuclease
COG5256	0.0490%	**0.1508%**	0.0520%	0.0214%	J	Translation elongation factor EF-1alpha (GTPase)
COG0532	0.1527%	**0.2846%**	0.1534%	0.1671%	J	Translation initiation factor 2 (IF-2; GTPase)
COG5257	0.0115%	**0.0584%**	0.0026%	0.0107%	J	Translation initiation factor 2, gamma subunit (eIF-2gamma; GTPase)
COG0290	0.0576%	**0.1776%**	0.0884%	0.0835%	J	Translation initiation factor 3 (IF-3)
COG4738	0.0115%	**0.0584%**	0.0026%	0.0000%	K	Predicted transcriptional regulator
COG3620	0.0000%	**0.0487%**	0.0000%	0.0021%	K	Predicted transcriptional regulator with C-terminal CBS domains
COG1695	0.0086%	**0.0705%**	0.0000%	0.0064%	K	Predicted transcriptional regulators
COG0640	0.0576%	**0.1289%**	0.0494%	0.0557%	K	Predicted transcriptional regulators
COG1777	0.0432%	**0.2433%**	0.0026%	0.0064%	K	Predicted transcriptional regulators
COG1405	0.0202%	**0.2311%**	0.0806%	0.0064%	K	Transcription initiation factor TFIIIB, Brf1 subunit/Transcription initiation factor TFIIB
COG1802	0.0000%	**0.0219%**	0.0000%	0.0021%	K	Transcriptional regulators
COG1846	0.0259%	**0.6009%**	0.0156%	0.0514%	K	Transcriptional regulators

COG	DBS-cDNA	FK-cDNA	YFS-cDNA	YFP-cDNA	COG category	COG annotation
**FK**						
COG0827	0.0115%	**0.0511%**	0.0078%	0.0000%	L	Adenine-specific DNA methylase
COG0507	0.0317%	**0.1265%**	0.0156%	0.0129%	L	ATP-dependent exoDNAse (exonuclease V), alpha subunit - helicase superfamily I member
COG0417	0.0000%	**0.0268%**	0.0000%	0.0021%	L	DNA polymerase elongation subunit (family B)
COG0582	0.3716%	**0.6982%**	0.3276%	0.2120%	L	Integrase
COG0322	0.0115%	**0.0657%**	0.0156%	0.0086%	L	Nuclease subunit of the excinuclease complex
COG1961	0.1037%	**0.3284%**	0.0260%	0.0278%	L	Site-specific recombinases, DNA invertase Pin homologs
COG5421	0.0202%	**0.1703%**	0.0208%	0.0150%	L	Transposase
COG3316	0.0029%	**0.0511%**	0.0000%	0.0021%	L	Transposase and inactivated derivatives
COG3547	0.0576%	**0.1216%**	0.0286%	0.0386%	L	Transposase and inactivated derivatives
COG3335	0.0375%	**0.1314%**	0.0364%	0.0343%	L	Transposase and inactivated derivatives
COG1943	0.0432%	**0.1533%**	0.0312%	0.0257%	L	Transposase and inactivated derivatives
COG3293	0.0058%	**0.1752%**	0.0026%	0.0129%	L	Transposase and inactivated derivatives
COG1686	0.0778%	**0.1776%**	0.0546%	0.0621%	M	D-alanyl-D-alanine carboxypeptidase
COG0449	0.0749%	**0.1533%**	0.0260%	0.0321%	M	Glucosamine 6-phosphate synthetase, contains amidotransferase and phosphosugar isomerase domains
COG0860	0.0288%	**0.6398%**	0.0390%	0.0343%	M	N-acetylmuramoyl-L-alanine amidase
COG2834	0.0346%	**0.1192%**	0.0130%	0.0600%	M	Outer membrane lipoprotein-sorting protein
COG2885	0.1988%	**0.6617%**	0.2184%	0.2763%	M	Outer membrane protein and related peptidoglycan-associated (lipo)proteins
COG0668	0.0086%	**0.0560%**	0.0052%	0.0000%	M	Small-conductance mechanosensitive channel
COG0773	0.0951%	**0.2871%**	0.0676%	0.0407%	M	UDP-N-acetylmuramate-alanine ligase
COG0638	0.0922%	**0.3600%**	0.1638%	0.0107%	O	20S proteasome, alpha and beta subunits
COG0719	0.1181%	**0.4768%**	0.1222%	0.1692%	O	ABC-type transport system involved in Fe-S cluster assembly, permease component
COG0464	0.0778%	**0.5863%**	0.1222%	0.0514%	O	ATPases of the AAA+ class
COG0465	0.3601%	**0.9196%**	0.5953%	0.4326%	O	ATP-dependent Zn proteases
COG1138	0.0317%	**0.1654%**	0.0286%	0.0450%	O	Cytochrome c biogenesis factor
COG2332	0.0029%	**0.1070%**	0.0052%	0.0150%	O	Cytochrome c-type biogenesis protein CcmE
COG0071	0.0691%	**0.4841%**	0.0780%	0.3320%	O	Molecular chaperone (small heat shock protein)

COG	DBS-cDNA	FK-cDNA	YFS-cDNA	YFP-cDNA	COG category	COG annotation
**FK**						
COG0225	0.0144%	**0.0949%**	0.0182%	0.0214%	O	Peptide methionine sulfoxide reductase
COG4930	0.0029%	**0.0389%**	0.0000%	0.0000%	O	Predicted ATP-dependent Lon-type protease
COG1067	0.0403%	**0.3333%**	0.0156%	0.0193%	O	Predicted ATP-dependent protease
COG1764	0.0029%	**0.0341%**	0.0052%	0.0000%	O	Predicted redox protein, regulator of disulfide bond formation
COG0492	0.0807%	**0.1995%**	0.0832%	0.0493%	O	Thioredoxin reductase
COG1612	0.0000%	**0.0462%**	0.0052%	0.0000%	O	Uncharacterized protein required for cytochrome oxidase assembly
COG1840	0.0000%	**0.0438%**	0.0078%	0.0000%	P	ABC-type Fe3+ transport system, periplasmic component
COG0600	0.0029%	**0.0462%**	0.0000%	0.0000%	P	ABC-type nitrate/sulfonate/bicarbonate transport system, permease component
COG0376	0.0144%	**0.0973%**	0.0052%	0.0043%	P	Catalase (peroxidase I)
COG2072	0.0029%	**0.1630%**	0.0000%	0.0043%	P	Predicted flavoprotein involved in K+ transport
COG2897	0.0086%	**0.0705%**	0.0052%	0.0021%	P	Rhodanese-related sulfurtransferase
COG0179	0.0115%	**0.0584%**	0.0026%	0.0043%	Q	2-keto-4-pentenoate hydratase/2-oxohepta-3-ene-1,7-dioic acid hydratase (catechol pathway)
COG4689	0.0000%	**0.0243%**	0.0000%	0.0000%	Q	Acetoacetate decarboxylase
COG3476	0.0000%	**0.0365%**	0.0000%	0.0000%	T	Tryptophan-rich sensory protein (mitochondrial benzodiazepine receptor homolog)
COG0745	0.1757%	**0.6374%**	0.1118%	0.2356%	TK	Response regulators consisting of a CheY-like receiver domain and a winged-helix DNA-binding domain
COG2948	0.0029%	**0.0389%**	0.0078%	0.0043%	U	Type IV secretory pathway, VirB10 components
COG0842	0.0547%	**0.1387%**	0.0130%	0.0236%	V	ABC-type multidrug transport system, permease component
COG1290	0.4206%	**0.1533%**	0.4991%	1.5313%	C	Cytochrome b subunit of the bc complex
COG2857	0.0951%	**0.0122%**	0.0806%	0.4476%	C	Cytochrome c1
COG2863	0.3543%	**0.0341%**	0.4160%	1.1544%	C	Cytochrome c553
COG0056	0.4984%	**0.1192%**	0.6291%	0.7111%	C	F0F1-type ATP synthase, alpha subunit
COG0055	0.3342%	**0.1557%**	0.2756%	0.4219%	C	F0F1-type ATP synthase, beta subunit
COG0712	0.2103%	**0.0122%**	0.1352%	0.1392%	C	F0F1-type ATP synthase, delta subunit
COG0437	0.0576%	**0.0024%**	0.0806%	0.0921%	C	Fe-S-cluster-containing hydrogenase components 1
COG1150	0.0893%	**0.0316%**	0.1508%	0.1585%	C	Heterodisulfide reductase, subunit C
COG3954	0.0893%	**0.0049%**	0.1014%	0.1092%	C	Phosphoribulokinase

COG	DBS-cDNA	FK-cDNA	YFS-cDNA	YFP-cDNA	COG category	COG annotation
**FK**						
COG5557	0.0403%	**0.0024%**	0.1872%	0.2163%	C	Polysulphide reductase
COG4451	0.5243%	**0.0195%**	0.5979%	1.7969%	C	Ribulose bisphosphate carboxylase small subunit
COG0002	0.1037%	**0.0316%**	0.1664%	0.0750%	E	Acetylglutamate semialdehyde dehydrogenase
COG1246	0.0403%	**0.0097%**	0.0416%	0.0600%	E	N-acetylglutamate synthase and related acetyltransferases
COG0458	0.2478%	**0.0754%**	0.1508%	0.1863%	EF	Carbamoylphosphate synthase large subunit (split gene in MJ)
COG0504	0.2478%	**0.0316%**	0.1300%	0.1028%	F	CTP synthase (UTP-ammonia lyase)
COG0519	0.1008%	**0.0389%**	0.0910%	0.1499%	F	GMP synthase, PP-ATPase domain/subunit
COG2301	0.2132%	**0.0024%**	0.2106%	0.0750%	G	Citrate lyase beta subunit
COG0191	0.2881%	**0.0024%**	0.2912%	0.1970%	G	Fructose/tagatose bisphosphate aldolase
COG0158	0.0951%	**0.0219%**	0.0858%	0.2035%	G	Fructose-1,6-bisphosphatase
COG3957	0.3572%	**0.0268%**	0.1508%	0.2313%	G	Phosphoketolase
COG2956	0.1008%	**0.0024%**	0.0416%	0.1563%	G	Predicted N-acetylglucosaminyl transferase
COG1850	2.0512%	**0.0511%**	1.4558%	2.7114%	G	Ribulose 1,5-bisphosphate carboxylase, large subunit
COG0021	0.1412%	**0.0414%**	0.2938%	0.1349%	G	Transketolase
COG1762	0.1152%	**0.0097%**	0.1040%	0.0493%	GT	Phosphotransferase system mannitol/fructose-specific IIA domain (Ntr-type)
COG0407	0.0979%	**0.0462%**	0.1092%	0.1414%	H	Uroporphyrinogen-III decarboxylase
COG3239	0.0432%	**0.0024%**	0.0624%	0.1349%	I	Fatty acid desaturase
COG0416	0.1901%	**0.0219%**	0.1378%	0.2270%	I	Fatty acid/phospholipid biosynthesis enzyme
COG1398	0.1757%	**0.0000%**	0.0936%	0.0171%	I	Fatty-acid desaturase
COG0304	0.3543%	**0.2311%**	0.3406%	0.4455%	IQ	3-oxoacyl-(acyl-carrier-protein) synthase
COG0236	0.1066%	**0.0268%**	0.0832%	0.0664%	IQ	Acyl carrier protein
COG0050	0.4523%	**0.2092%**	0.4783%	1.1929%	J	GTPases - translation elongation factors
COG0016	0.2218%	**0.0146%**	0.0624%	0.2077%	J	Phenylalanyl-tRNA synthetase alpha subunit
COG0081	0.1354%	**0.0608%**	0.1196%	0.2527%	J	Ribosomal protein L1
COG0244	0.2103%	**0.1095%**	0.2002%	0.7924%	J	Ribosomal protein L10
COG0080	0.2478%	**0.0632%**	0.1222%	0.2592%	J	Ribosomal protein L11

COG	DBS-cDNA	FK-cDNA	YFS-cDNA	YFP-cDNA	COG category	COG annotation
**FK**						
COG0090	0.5618%	**0.1946%**	0.4160%	0.5890%	J	Ribosomal protein L2
COG0261	0.0778%	**0.0292%**	0.0858%	0.1392%	J	Ribosomal protein L21
COG0255	0.0720%	**0.0073%**	0.0572%	0.1157%	J	Ribosomal protein L29
COG0222	0.3169%	**0.1192%**	0.2340%	0.5097%	J	Ribosomal protein L7/L12
COG0051	0.2852%	**0.0876%**	0.2288%	0.8503%	J	Ribosomal protein S10
COG0099	0.2045%	**0.0341%**	0.1976%	0.4112%	J	Ribosomal protein S13
COG0199	0.1210%	**0.0219%**	0.0910%	0.1456%	J	Ribosomal protein S14
COG0185	0.2276%	**0.0827%**	0.1950%	0.2977%	J	Ribosomal protein S19
COG0049	0.2391%	**0.1070%**	0.1976%	0.2977%	J	Ribosomal protein S7
COG0096	0.6770%	**0.0876%**	0.9281%	0.3684%	J	Ribosomal protein S8
COG0480	0.6251%	**0.4792%**	0.6785%	0.6811%	J	Translation elongation factors (GTPases)
COG0361	0.0691%	**0.0146%**	0.0858%	0.0750%	J	Translation initiation factor 1 (IF-1)
COG0085	1.2042%	**0.4598%**	0.9645%	1.4971%	K	DNA-directed RNA polymerase, beta subunit/140 kD subunit
COG1396	0.0663%	**0.0195%**	0.0624%	0.0578%	K	Predicted transcriptional regulators
COG0305	0.2161%	**0.0268%**	0.0728%	0.1157%	L	Replicative DNA helicase
COG0513	0.2996%	**0.0341%**	0.1014%	0.1221%	LKJ	Superfamily II DNA and RNA helicases
COG1043	0.0519%	**0.0024%**	0.0338%	0.0321%	M	Acyl-[acyl carrier protein]--UDP-N-acetylglucosamine O-acyltransferase
COG0791	0.0173%	**0.0000%**	0.0208%	0.0750%	M	Cell wall-associated hydrolases (invasion-associated proteins)
COG2825	0.0259%	**0.0000%**	0.0312%	0.0214%	M	Outer membrane protein
COG1596	0.0432%	**0.0049%**	0.0520%	0.0407%	M	Periplasmic protein involved in polysaccharide export
COG1345	0.0634%	**0.0049%**	0.0988%	0.0321%	N	Flagellar capping protein
COG0326	0.1325%	**0.0462%**	0.4264%	1.2187%	O	Molecular chaperone, HSP90 family
COG1765	0.0375%	**0.0073%**	0.0364%	0.0343%	O	Predicted redox protein, regulator of disulfide bond formation
COG1333	0.1527%	**0.0730%**	0.1352%	0.4905%	O	ResB protein required for cytochrome c biosynthesis
COG0226	0.7577%	**0.2068%**	0.5641%	1.9576%	P	ABC-type phosphate transport system, periplasmic component
COG2895	0.0663%	**0.0073%**	0.0364%	0.0493%	P	GTPases - Sulfate adenylate transferase subunit 1

COG	DBS-cDNA	FK-cDNA	YFS-cDNA	YFP-cDNA	COG category	COG annotation
**FK**						
COG4548	0.2189%	**0.0146%**	0.2158%	0.3812%	P	Nitric oxide reductase activation protein
COG0848	0.2794%	**0.0657%**	0.1716%	0.5740%	U	Biopolymer transport protein
COG0342	0.1354%	**0.0414%**	0.0910%	0.1542%	U	Preprotein translocase subunit SecD
COG3023	0.0288%	**0.0000%**	0.0208%	0.0214%	V	Negative regulator of beta-lactamase expression
**YFS**						
COG1048	0.1844%	0.4671%	**0.6525%**	0.0835%	C	Aconitase A
COG2993	0.0461%	0.1387%	**0.7227%**	0.0750%	C	Cbb3-type cytochrome oxidase, cytochrome c subunit
COG2221	0.0086%	0.0146%	**0.0676%**	0.0021%	C	Dissimilatory sulfite reductase (desulfoviridin), alpha and beta subunits
COG0114	0.0144%	0.0146%	**0.0624%**	0.0129%	C	Fumarase
COG1740	0.0058%	0.0122%	**0.0494%**	0.0000%	C	Ni,Fe-hydrogenase I small subunit
COG0822	0.0691%	0.2360%	**0.4134%**	0.0386%	C	NifU homolog involved in Fe-S cluster formation
COG5016	0.0000%	0.0024%	**0.0286%**	0.0000%	C	Pyruvate/oxaloacetate carboxyltransferase
COG0674	0.1412%	0.5109%	**1.1569%**	0.0471%	C	Pyruvate:ferredoxin oxidoreductase and related 2-oxoacid:ferredoxin oxidoreductases, alpha subunit
COG1144	0.0691%	0.0584%	**0.3666%**	0.0043%	C	Pyruvate:ferredoxin oxidoreductase and related 2-oxoacid:ferredoxin oxidoreductases, delta subunit
COG1014	0.1613%	0.4963%	**1.0191%**	0.0535%	C	Pyruvate:ferredoxin oxidoreductase and related 2-oxoacid:ferredoxin oxidoreductases, gamma subunit
COG0074	0.1844%	0.2092%	**1.0035%**	0.0814%	C	Succinyl-CoA synthetase, alpha subunit
COG0045	0.0663%	0.1873%	**0.3016%**	0.0942%	C	Succinyl-CoA synthetase, beta subunit
COG0473	0.0663%	0.0973%	**0.8371%**	0.0171%	CE	Isocitrate/isopropylmalate dehydrogenase
COG1052	0.0691%	0.0365%	**0.1404%**	0.0171%	CHR	Lactate dehydrogenase and related dehydrogenases
COG0065	0.0259%	0.0219%	**0.0936%**	0.0278%	E	3-isopropylmalate dehydratase large subunit
COG0747	0.0317%	0.2700%	**0.5329%**	0.0621%	E	ABC-type dipeptide transport system, periplasmic component
COG1104	0.1440%	0.3187%	**0.6863%**	0.0728%	E	Cysteine sulfinate desulfinase/cysteine desulfurase and related enzymes
COG0174	0.2679%	0.4087%	**0.8969%**	0.3598%	E	Glutamine synthetase
COG0059	0.1498%	0.1606%	**0.2912%**	0.1456%	EH	Ketol-acid reductoisomerase
COG0034	0.0720%	0.1168%	**0.2496%**	0.0343%	F	Glutamine phosphoribosylpyrophosphate amidotransferase
COG0362	0.0000%	0.0000%	**0.0286%**	0.0000%	G	6-phosphogluconate dehydrogenase

COG	DBS-cDNA	FK-cDNA	YFS-cDNA	YFP-cDNA	COG category	COG annotation
**YFS**						
COG0364	0.0576%	0.0730%	**0.1456%**	0.0643%	G	Glucose-6-phosphate 1-dehydrogenase
COG0057	0.0634%	0.0754%	**0.1768%**	0.0578%	G	Glyceraldehyde-3-phosphate dehydrogenase/erythrose-4-phosphate dehydrogenase
COG1023	0.0259%	0.0292%	**0.1092%**	0.0300%	G	Predicted 6-phosphogluconate dehydrogenase
COG0021	0.1412%	0.0414%	**0.2938%**	0.1349%	G	Transketolase
COG0190	0.0058%	0.0097%	**0.0468%**	0.0021%	H	5,10-methylene-tetrahydrofolate dehydrogenase/Methenyl tetrahydrofolate cyclohydrolase
COG0351	0.0058%	0.0073%	**0.0390%**	0.0043%	H	Hydroxymethylpyrimidine/phosphomethylpyrimidine kinase
COG0422	0.1296%	0.1679%	**0.5121%**	0.0450%	H	Thiamine biosynthesis protein ThiC
COG0761	0.0432%	0.1825%	**0.4393%**	0.0514%	IM	Penicillin tolerance protein
COG0154	0.0230%	0.0097%	**0.0676%**	0.0214%	J	Asp-tRNAAsn/Glu-tRNAGln amidotransferase A subunit and related amidases
COG0096	0.6770%	0.0876%	**0.9281%**	0.3684%	J	Ribosomal protein S8
COG0178	0.0202%	0.0292%	**0.0962%**	0.0150%	L	Excinuclease ATPase subunit
COG3774	0.0029%	0.0000%	**0.0650%**	0.0214%	M	Mannosyltransferase OCH1 and related enzymes
COG0562	0.0058%	0.0097%	**0.0546%**	0.0150%	M	UDP-galactopyranose mutase
COG1344	0.2823%	0.5595%	**0.9957%**	0.0428%	N	Flagellin and related hook-associated proteins
COG1516	0.0086%	0.0024%	**0.0520%**	0.0000%	NUO	Flagellin-specific chaperone FliS
COG0542	0.5214%	1.2504%	**1.5208%**	0.7903%	O	ATPases with chaperone activity, ATP-binding subunit
COG3278	0.1815%	0.4817%	**3.0338%**	0.3684%	O	Cbb3-type cytochrome oxidase, subunit 1
COG0443	0.3918%	0.8393%	**1.3492%**	0.8396%	O	Molecular chaperone
COG0652	0.0490%	0.0560%	**0.1092%**	0.0364%	O	Peptidyl-prolyl cis-trans isomerase (rotamase) - cyclophilin family
COG0725	0.0375%	0.0243%	**0.1170%**	0.0578%	P	ABC-type molybdate transport system, periplasmic component
COG0004	0.0605%	0.0803%	**0.1846%**	0.0643%	P	Ammonia permease
COG2046	0.0058%	0.0146%	**0.0520%**	0.0129%	P	ATP sulfurylase (sulfate adenylyltransferase)
COG0672	0.0029%	0.0438%	**0.5589%**	0.0064%	P	High-affinity Fe2+/Pb2+ permease
COG0412	0.0288%	0.0535%	**0.1092%**	0.0129%	Q	Dienelactone hydrolase and related enzymes
COG2931	0.0086%	0.0024%	**0.0468%**	0.0107%	Q	RTX toxins and related Ca2+-binding proteins
COG5295	0.0058%	0.0000%	**0.0962%**	0.0129%	UW	Autotransporter adhesin

COG	DBS-cDNA	FK-cDNA	YFS-cDNA	YFP-cDNA	COG category	COG annotation
**YFS**						
COG0281	0.0519%	0.0705%	**0.0130%**	0.0578%	C	Malic enzyme
COG0838	0.0519%	0.0754%	**0.0000%**	0.0621%	C	NADH:ubiquinone oxidoreductase subunit 3 (chain A)
COG1071	0.0720%	0.0949%	**0.0182%**	0.1049%	C	Pyruvate/2-oxoglutarate dehydrogenase complex, dehydrogenase (E1) component, eukaryotic type, alpha subunit
COG1192	0.1066%	0.1606%	**0.0390%**	0.1071%	D	ATPases involved in chromosome partitioning
COG0685	0.1008%	0.0657%	**0.0104%**	0.0621%	E	5,10-methylenetetrahydrofolate reductase
COG0563	0.0922%	0.1119%	**0.0156%**	0.0600%	F	Adenylate kinase and related kinases
COG1134	0.0605%	0.1168%	**0.0078%**	0.0364%	GM	ABC-type polysaccharide/polyol phosphate transport system, ATPase component
COG1278	0.1844%	0.1362%	**0.0520%**	0.1178%	K	Cold shock proteins
COG0776	0.2506%	0.1606%	**0.0884%**	0.3834%	L	Bacterial nucleoid DNA-binding protein
COG3666	0.0317%	0.0243%	**0.0000%**	0.0578%	L	Transposase and inactivated derivatives
COG0438	0.2478%	0.2384%	**0.1170%**	0.2120%	M	Glycosyltransferase
COG1210	0.0461%	0.0730%	**0.0130%**	0.0643%	M	UDP-glucose pyrophosphorylase
COG0625	0.1210%	0.1411%	**0.0598%**	0.1199%	O	Glutathione S-transferase
COG0605	0.1613%	0.1435%	**0.0520%**	0.2120%	P	Superoxide dismutase
COG2146	0.0317%	0.0632%	**0.0026%**	0.0343%	PR	Ferredoxin subunits of nitrite reductase and ring-hydroxylating dioxygenases
COG0642	0.1873%	0.1143%	**0.0468%**	0.1606%	T	Signal transduction histidine kinase
COG0811	0.2305%	0.2700%	**0.1040%**	0.4005%	U	Biopolymer transport proteins
**YFP**						
COG0372	0.1786%	0.1630%	0.1846%	**0.3106%**	C	Citrate synthase
COG1290	0.4206%	0.1533%	0.4991%	**1.5313%**	C	Cytochrome b subunit of the bc complex
COG3258	0.0720%	0.0973%	0.1170%	**0.2120%**	C	Cytochrome c
COG2857	0.0951%	0.0122%	0.0806%	**0.4476%**	C	Cytochrome c1
COG2863	0.3543%	0.0341%	0.4160%	**1.1544%**	C	Cytochrome c553
COG0224	0.1037%	0.0584%	0.0962%	**0.3705%**	C	F0F1-type ATP synthase, gamma subunit
COG0843	0.0749%	0.5133%	0.2028%	**1.5292%**	C	Heme/copper-type cytochrome/quinol oxidases, subunit 1
COG1622	0.0922%	0.1314%	0.1014%	**1.0644%**	C	Heme/copper-type cytochrome/quinol oxidases, subunit 2

COG	DBS-cDNA	FK-cDNA	YFS-cDNA	YFP-cDNA	COG category	COG annotation
**YFP**						
COG4451	0.5243%	0.0195%	0.5979%	**1.7969%**	C	Ribulose bisphosphate carboxylase small subunit
COG3705	0.0115%	0.0097%	0.0130%	**0.0600%**	E	ATP phosphoribosyltransferase involved in histidine biosynthesis
COG0509	0.0576%	0.0365%	0.0520%	**0.2592%**	E	Glycine cleavage system H protein (lipoate-binding)
COG0158	0.0951%	0.0219%	0.0858%	**0.2035%**	G	Fructose-1,6-bisphosphatase
COG1850	2.0512%	0.0511%	1.4558%	**2.7114%**	G	Ribulose 1,5-bisphosphate carboxylase, large subunit
COG0314	0.0230%	0.0097%	0.0312%	**0.1371%**	H	Molybdopterin converting factor, large subunit
COG0543	0.1383%	0.0803%	0.1014%	**0.2270%**	HC	2-polyprenylphenol hydroxylase and related flavodoxin oxidoreductases
COG3239	0.0432%	0.0024%	0.0624%	**0.1349%**	I	Fatty acid desaturase
COG1028	0.4379%	0.3138%	0.2262%	**0.9102%**	IQR	Dehydrogenases with different specificities (related to short-chain alcohol dehydrogenases)
COG0050	0.4523%	0.2092%	0.4783%	**1.1929%**	J	GTPases - translation elongation factors
COG0081	0.1354%	0.0608%	0.1196%	**0.2527%**	J	Ribosomal protein L1
COG0244	0.2103%	0.1095%	0.2002%	**0.7924%**	J	Ribosomal protein L10
COG0292	0.1210%	0.0949%	0.1040%	**0.2077%**	J	Ribosomal protein L20
COG0089	0.1210%	0.0681%	0.0806%	**0.2763%**	J	Ribosomal protein L23
COG0211	0.1383%	0.2019%	0.2054%	**0.3341%**	J	Ribosomal protein L27
COG0087	0.3572%	0.2238%	0.2652%	**0.6682%**	J	Ribosomal protein L3
COG1841	0.0058%	0.0122%	0.0286%	**0.1007%**	J	Ribosomal protein L30/L7E
COG0222	0.3169%	0.1192%	0.2340%	**0.5097%**	J	Ribosomal protein L7/L12
COG0051	0.2852%	0.0876%	0.2288%	**0.8503%**	J	Ribosomal protein S10
COG0099	0.2045%	0.0341%	0.1976%	**0.4112%**	J	Ribosomal protein S13
COG0186	0.0922%	0.0535%	0.0780%	**0.2399%**	J	Ribosomal protein S17
COG0092	0.2161%	0.0632%	0.0858%	**0.4026%**	J	Ribosomal protein S3
COG0566	0.0086%	0.0000%	0.0026%	**0.0535%**	J	rRNA methylases
COG0085	1.2042%	0.4598%	0.9645%	**1.4971%**	K	DNA-directed RNA polymerase, beta subunit/140 kD subunit
COG0557	0.0634%	0.0170%	0.0416%	**0.2185%**	K	Exoribonuclease R
COG3561	0.0029%	0.0000%	0.0026%	**0.0557%**	K	Phage anti-repressor protein

COG	DBS-cDNA	FK-cDNA	YFS-cDNA	YFP-cDNA	COG category	COG annotation
**YFP**						
COG1386	0.0000%	0.0000%	0.0000%	**0.0343%**	K	Predicted transcriptional regulator containing the HTH domain
COG0250	0.0490%	0.0560%	0.0468%	**0.1178%**	K	Transcription antiterminator
COG0776	0.2506%	0.1606%	0.0884%	**0.3834%**	L	Bacterial nucleoid DNA-binding protein
COG0708	0.0144%	0.0195%	0.0130%	**0.2099%**	L	Exonuclease III
COG1193	0.0288%	0.0049%	0.0130%	**0.0771%**	L	Mismatch repair ATPase (MutS family)
COG0791	0.0173%	0.0000%	0.0208%	**0.0750%**	M	Cell wall-associated hydrolases (invasion-associated proteins)
COG2943	0.0893%	0.0097%	0.0130%	**0.2934%**	M	Membrane glycosyltransferase
COG3511	0.0403%	0.0097%	0.0312%	**0.1499%**	M	Phospholipase C
COG1004	0.0432%	0.0365%	0.0546%	**0.1328%**	M	Predicted UDP-glucose 6-dehydrogenase
COG3170	0.0230%	0.0170%	0.0052%	**0.0750%**	NU	Tfp pilus assembly protein FimV
COG0755	0.3515%	0.3041%	0.3146%	**1.2187%**	O	ABC-type transport system involved in cytochrome c biogenesis, permease component
COG0466	0.2189%	0.2895%	0.3432%	**0.8053%**	O	ATP-dependent Lon protease, bacterial type
COG0234	0.4062%	0.1873%	0.1430%	**0.6532%**	O	Co-chaperonin GroES (HSP10)
COG0330	0.1786%	0.1168%	0.1300%	**0.3084%**	O	Membrane protease subunits, stomatin/prohibitin homologs
COG0326	0.1325%	0.0462%	0.4264%	**1.2187%**	O	Molecular chaperone, HSP90 family
COG1333	0.1527%	0.0730%	0.1352%	**0.4905%**	O	ResB protein required for cytochrome c biosynthesis
COG0226	0.7577%	0.2068%	0.5641%	**1.9576%**	P	ABC-type phosphate transport system, periplasmic component
COG0581	0.0691%	0.0219%	0.0130%	**0.2077%**	P	ABC-type phosphate transport system, permease component
COG2193	0.0173%	0.0049%	0.0598%	**0.2099%**	P	Bacterioferritin (cytochrome b1)
COG4548	0.2189%	0.0146%	0.2158%	**0.3812%**	P	Nitric oxide reductase activation protein
COG0369	0.0778%	0.0316%	0.0078%	**0.2292%**	P	Sulfite reductase, alpha subunit (flavoprotein)
COG4454	0.0634%	0.0024%	0.0052%	**0.1478%**	P	Uncharacterized copper-binding protein
COG4577	0.0144%	0.0049%	0.0156%	**0.1135%**	QC	Carbon dioxide concentrating mechanism/carboxysome shell protein
COG0848	0.2794%	0.0657%	0.1716%	**0.5740%**	U	Biopolymer transport protein
COG0811	0.2305%	0.2700%	0.1040%	**0.4005%**	U	Biopolymer transport proteins
COG1048	0.1844%	0.4671%	0.6525%	**0.0835%**	C	Aconitase A

COG	DBS-cDNA	FK-cDNA	YFS-cDNA	YFP-cDNA	COG category	COG annotation
**YFP**						
COG0356	0.3083%	0.1727%	0.1794%	**0.0835%**	C	F0F1-type ATP synthase, subunit a
COG1145	0.1181%	0.1970%	0.2366%	**0.0107%**	C	Ferredoxin
COG3411	0.0547%	0.0730%	0.0338%	**0.0064%**	C	Ferredoxin
COG0538	0.1527%	0.1022%	0.1638%	**0.0471%**	C	Isocitrate dehydrogenases
COG0039	0.0749%	0.1070%	0.0702%	**0.0236%**	C	Malate/lactate dehydrogenases
COG1894	0.3227%	0.7979%	0.5537%	**0.0900%**	C	NADH:ubiquinone oxidoreductase, NADH-binding (51 kD) subunit
COG0674	0.1412%	0.5109%	1.1569%	**0.0471%**	C	Pyruvate:ferredoxin oxidoreductase and related 2-oxoacid:ferredoxin oxidoreductases, alpha subunit
COG1013	0.1729%	0.5328%	0.5069%	**0.0471%**	C	Pyruvate:ferredoxin oxidoreductase and related 2-oxoacid:ferredoxin oxidoreductases, beta subunit
COG1144	0.0691%	0.0584%	0.3666%	**0.0043%**	C	Pyruvate:ferredoxin oxidoreductase and related 2-oxoacid:ferredoxin oxidoreductases, delta subunit
COG1014	0.1613%	0.4963%	1.0191%	**0.0535%**	C	Pyruvate:ferredoxin oxidoreductase and related 2-oxoacid:ferredoxin oxidoreductases, gamma subunit
COG0479	0.0317%	0.0560%	0.0260%	**0.0021%**	C	Succinate dehydrogenase/fumarate reductase, Fe-S protein subunit
COG0074	0.1844%	0.2092%	1.0035%	**0.0814%**	C	Succinyl-CoA synthetase, alpha subunit
COG0473	0.0663%	0.0973%	0.8371%	**0.0171%**	CE	Isocitrate/isopropylmalate dehydrogenase
COG0165	0.0490%	0.0632%	0.0520%	**0.0086%**	E	Argininosuccinate lyase
COG1104	0.1440%	0.3187%	0.6863%	**0.0728%**	E	Cysteine sulfinate desulfinase/cysteine desulfurase and related enzymes
COG0031	0.0230%	0.0487%	0.0338%	**0.0021%**	E	Cysteine synthase
COG1003	0.0519%	0.0997%	0.0520%	**0.0064%**	E	Glycine cleavage system protein P (pyridoxal-binding), C-terminal domain
COG1509	0.0346%	0.0462%	0.0572%	**0.0021%**	E	Lysine 2,3-aminomutase
COG0493	0.2161%	0.2506%	0.2366%	**0.1092%**	ER	NADPH-dependent glutamate synthase beta chain and related oxidoreductases
COG0448	0.1700%	0.0924%	0.1638%	**0.0450%**	G	ADP-glucose pyrophosphorylase
COG0113	0.0605%	0.1095%	0.0520%	**0.0171%**	H	Delta-aminolevulinic acid dehydratase
COG0142	0.2103%	0.1970%	0.1716%	**0.0600%**	H	Geranylgeranyl pyrophosphate synthase
COG2226	0.0634%	0.0924%	0.0572%	**0.0193%**	H	Methylase involved in ubiquinone/menaquinone biosynthesis
COG1995	0.0490%	0.0438%	0.0494%	**0.0043%**	H	Pyridoxal phosphate biosynthesis protein
COG0422	0.1296%	0.1679%	0.5121%	**0.0450%**	H	Thiamine biosynthesis protein ThiC
COG0111	0.0461%	0.1070%	0.1222%	**0.0064%**	HE	Phosphoglycerate dehydrogenase and related dehydrogenases

COG	DBS-cDNA	FK-cDNA	YFS-cDNA	YFP-cDNA	COG category	COG annotation
**YFP**						
COG2084	0.0634%	0.0705%	0.1196%	**0.0193%**	I	3-hydroxyisobutyrate dehydrogenase and related beta-hydroxyacid dehydrogenases
COG0777	0.1181%	0.0827%	0.1326%	**0.0386%**	I	Acetyl-CoA carboxylase beta subunit
COG0495	0.0375%	0.0754%	0.0416%	**0.0064%**	J	Leucyl-tRNA synthetase
COG0828	0.1671%	0.0389%	0.0468%	**0.0064%**	J	Ribosomal protein S21
COG0172	0.0346%	0.0705%	0.0390%	**0.0021%**	J	Seryl-tRNA synthetase
COG3437	0.0403%	0.0292%	0.0598%	**0.0043%**	KT	Response regulator containing a CheY-like receiver domain and an HD-GYP domain
COG0582	0.3716%	0.6982%	0.3276%	**0.2120%**	L	Integrase
COG0164	0.0375%	0.0341%	0.0260%	**0.0021%**	L	Ribonuclease HII
COG0675	1.4347%	0.3333%	0.2938%	**0.1542%**	L	Transposase and inactivated derivatives
COG0768	0.1210%	0.2262%	0.1482%	**0.0321%**	M	Cell division protein FtsI/penicillin-binding protein 2
COG1181	0.0432%	0.0292%	0.0598%	**0.0043%**	M	D-alanine-D-alanine ligase and related ATP-grasp enzymes
COG1360	0.0346%	0.0219%	0.0260%	**0.0000%**	N	Flagellar motor protein
COG1344	0.2823%	0.5595%	0.9957%	**0.0428%**	N	Flagellin and related hook-associated proteins
COG0840	0.1527%	0.1460%	0.1326%	**0.0236%**	NT	Methyl-accepting chemotaxis protein
COG0638	0.0922%	0.3600%	0.1638%	**0.0107%**	O	20S proteasome, alpha and beta subunits
COG0526	0.1757%	0.3527%	0.4056%	**0.1007%**	OC	Thiol-disulfide isomerase and thioredoxins
COG2200	0.0634%	0.0705%	0.0884%	**0.0236%**	T	FOG: EAL domain
COG1217	0.2622%	0.1654%	0.1690%	**0.0857%**	T	Predicted membrane GTPase involved in stress response
COG1136	0.0403%	0.0487%	0.0676%	**0.0107%**	V	ABC-type antimicrobial peptide transport system, ATPase component
COG1403	0.4206%	0.3284%	0.1300%	**0.0621%**	V	Restriction endonuclease

The relative abundance of the COGs with significantly higher and lower expression levels in one community than those in all other three communities are shown. The relative abundance was calculated as the number of cDNA protein-coding sequences assigned to the COG in a community divided by the total number of cDNA protein-coding sequences in the community. The COGs in red and blue represented those with significantly higher and lower expression levels in the community, respectively. For each community, the COGs were ordered according to their assignment to COG categories.
