# Supplementary material for: Comparative metagenomic and metatranscriptomic analyses of microbial communities in acid mine drainage
Source: ISME J. 2014 Dec 23;9(7):1579–92. doi: 10.1038/ismej.2014.245 (PMC4478699; doi:10.1038/ismej.2014.245)
Supplement: Supplementary Table 4 [file ismej2014245x4.pdf]

Table S4 Detailed information of genes with significantly different expression activities in *At. ferrivorans* in DBS, YFS and YFP

Functions	NCBI ID	Gene annotation	Relative abundance (%)			*P* value *		
			DBS	YFS	YFP	DBS/YFS	DBS/YFP	YFS/YFP
Ribosomal proteins	YP_004785088.1	30S ribosomal protein S11	0.03	0.00	0.14		0.032	
	YP_004785051.1	30S ribosomal protein S15	0.04	0.29	0.17	0.014	0.017	
	YP_004784808.1	30S ribosomal protein S21	0.00	0.15	0.01	0.033		0.041
	YP_004785123.1	50S ribosomal protein L1	0.09	0.04	0.31		0.002	0.091
	YP_004785122.1	50S ribosomal protein L10	0.34	0.73	0.92		0.000	
	YP_004785124.1	50S ribosomal protein L11	0.46	0.07	0.27	0.033		
	YP_004785485.1	50S ribosomal protein L13	0.82	0.36	0.45		0.001	
	YP_004785108.1	50S ribosomal protein L22	0.06	0.11	0.23		0.010	
	YP_004783734.1	50S ribosomal protein L25	0.14	0.04	0.01		0.000	
	YP_004785111.1	50S ribosomal protein L25/L23	0.11	0.07	0.27		0.045	
	YP_004785149.1	50S ribosomal protein L27	0.09	0.18	0.25		0.040	
	YP_004785113.1	50S ribosomal protein L3	0.55	0.55	1.05		0.000	
	YP_004785098.1	50S ribosomal protein L6	0.10	0.22	0.36		0.000	
	YP_004785121.1	50S ribosomal protein L7/L12	0.13	0.11	0.45		0.000	0.094
RNA polymerase	YP_004785086.1	DNA-directed RNA polymerase subunit alpha	0.02	0.11	0.21		0.000	
	YP_004785120.1	DNA-directed RNA polymerase subunit beta	1.85	1.56	1.33		0.009	
	YP_004785119.1	DNA-directed RNA polymerase subunit beta\'	0.71	0.15	0.27	0.005	0.000	
Chaperonins	YP_004784720.1	60 kDa chaperonin	0.29	2.98	1.58	0.000	0.000	0.000
	YP_004783122.1	chaperone protein dnaK	0.04	0.80	0.69	0.000	0.000	
	YP_004784036.1	chaperone protein htpG	0.03	0.15	0.21		0.000	
rus operon	YP_004783009.1	rusticyanin	0.60	0.47	0.24		0.000	
	YP_004783524.1	cytochrome c oxidase subunit I	0.01	0.00	0.01			
	YP_004784176.1	rusticyanin	0.25	0.29	0.36			
	YP_004784314.1	rusticyanin	5.30	2.22	6.41	0.000	0.002	0.000
	YP_004784315.1	hypothetical protein Acife_1865	0.00	0.04	0.02			
	YP_004784317.1	cytochrome c oxidase, aa3-type subunit III	0.01	0.18	0.16	0.033	0.000	
	YP_004784318.1	cytochrome c oxidase subunit I	0.10	0.55	1.74	0.001	0.000	0.000
	YP_004784319.1	cytochrome c oxidase subunit II	0.13	0.33	0.98		0.000	0.007
	YP_004784320.1	hypothetical protein Acife_1870	0.24	0.25	0.33			
	YP_004784321.1	cytochrome c class I	0.21	0.66	0.55	0.016	0.000	
	YP_004784322.1	cytochrome c	0.21	0.84	1.44	0.000	0.000	
Sulfur oxidation	YP_004782606.1	pyrrolo-quinoline quinone repeat-containing protein	0.02	0.00	0.03			
	YP_004783513.1	cytochrome c oxidase subunit III	0.00	0.00	0.02			
	YP_004783859.1	periplasmic solute-binding protein	0.03	0.04	0.03			
	YP_004783860.1	TQO small subunit DoxD domain-containing protein	0.04	0.18	0.03			
	YP_004784909.1	hypothetical protein Acife_2490	0.00	0.04	0.01			
	YP_004784910.1	sulfur oxidation protein SoxZ	0.01	0.07	0.00			
	YP_004784912.1	5\'-nucleotidase domain-containing protein	0.03	0.04	0.03			
	YP_004785010.1	pyrrolo-quinoline quinone repeat-containing protein	0.00	0.29	0.00	0.000		0.000
	YP_004785011.1	Tat pathway signal sequence domain-containing protein	0.00	0.00	0.00			
CBB carbon fixation cycle	YP_004784671.1	ribulose bisphosphate carboxylase large chain	1.92	2.22	1.15		0.000	0.001
	YP_004785539.1	ribulose bisphosphate carboxylase large chain	0.42	1.35	0.99	0.000	0.000	
	YP_004785538.1	ribulose bisphosphate carboxylase small chain	0.04	1.82	0.26	0.000	0.000	0.000
NADH dehydrogenase	YP_004784326.1	NADH/Ubiquinone/plastoquinone (complex I)	0.00	1.38	0.05	0.000		0.000
	YP_004782988.1	NADH/Ubiquinone/plastoquinone (complex I)	0.05	0.44	0.17	0.001	0.085	
Carboxysomes	YP_004785536.1	carboxysome shell carbonic anhydrase	0.06	1.35	0.10	0.000		0.000
	YP_004785537.1	carboxysome structural protein CsoS2	0.02	1.20	0.22	0.000	0.000	0.000
Glutamate synthesis	YP_004784608.1	ferredoxin-dependent glutamate synthase	0.14	0.40	0.06			0.001
	YP_004783365.1	glutamine synthetase, type I	0.13	0.62	0.39	0.001	0.000	
Phosphate assimilation	YP_004783991.1	phosphate ABC transporter substrate-binding protein	1.11	4.62	2.66	0.000	0.000	0.000
	YP_004783099.1	phosphate-selective porin O and P	0.08	2.22	0.91	0.000	0.000	0.000
Assimilatory sulfur reduction	YP_004785029.1	flavodoxin/nitric oxide synthase	0.31	0.00	0.35	0.022		0.006
	YP_004785030.1	sulfite reductase (NADPH) hemoprotein beta-component	0.48	0.04	0.15	0.004	0.000	
	YP_004785033.1	sulfate adenylyltransferase large subunit	0.07	0.00	0.00		0.027	
	YP_004785032.1	sulfate adenylyltransferase small subunit	0.12	0.00	0.01		0.005	

*Only those P value ≤ 0.05 are shown.
