# Supplementary material for: Comparative metagenomic and metatranscriptomic analyses of microbial communities in acid mine drainage
Source: ISME J. 2014 Dec 23;9(7):1579–92. doi: 10.1038/ismej.2014.245 (PMC4478699; doi:10.1038/ismej.2014.245)
Supplement: Supplementary Table 5 [file ismej2014245x5.pdf]

Table S5 Detailed information of genes with significantly different expression activities in *L. ferrodiazotrophum* in the communities of DBS, FK and YFS

Functions	NCBI ID	Gene annotation	Relative abundance (%)			*P* value*		
			DBS	YFS	YFP	DBS-FK	DBS-YFS	FK-YFS
Nitrogen fixation	EES53481.1	conserved hypothetical protein	0.000	0.000	0.000			
	EES53482.1	conserved hypothetical protein	0.000	0.000	0.000			
	EES53483.1	hypothetical protein UBAL3_78920076	0.000	0.000	0.029			
	EES53484.1	Nitrogenase iron protein (NifH)	0.012	0.000	0.058			
	EES53485.1	Nitrogenase, molybdenum-iron protein alpha chain (NifD)	0.000	0.018	0.145		0.0468	0.0680
	EES53486.1	Nitrogenase, molybdenum-iron protein beta chain (NifK)	0.000	0.000	0.000			
	EES53487.1	Nitrogenase MoFe cofactor biosynthesis protein (NifE)	0.000	0.000	0.000			
	EES53488.1	Nitrogenase molybdenum-iron cofactor biosynthesis protein (NifN)	0.000	0.005	0.000			
	EES53489.1	Nitrogenase molybdenum-iron protein (NifX)	0.000	0.000	0.000			
	EES53490.1	NifX-associated protein	0.000	0.005	0.000			
	EES53491.1	probable transposase, partial	0.000	0.000	0.000			
	EES53492.1	Nitrogenase cofactor biosynthesis protein (NifB)	0.000	0.000	0.000			
	EES53493.1	putative ferredoxin	0.012	0.000	0.000			
	EES53494.1	probable iron-sulfur cluster assembly family protei	0.000	0.000	0.000			
	EES53495.1	conserved hypothetical protein	0.000	0.000	0.000			
	EES53496.1	conserved hypothetical protein	0.000	0.000	0.029			
rTCA carbon fixation	EES51678.1	isocitrate dehydrogenase, NADP-dependent	0.204	0.032	0.087	0.0008		
	EES52178.1	phosphoenolpyruvate synthase	0.096	0.110	0.058			
	EES52748.1	Succinyl-CoA synthetase, beta subunit	0.060	0.078	0.233			
	EES52749.1	Succinyl-CoA synthetase, alpha subunit	0.516	0.205	0.378	0.0012		
	EES52750.1	putative aconitate hydratase	0.384	0.388	0.640			
	EES52751.1	probable citrate synthase	0.108	0.073	0.291			0.0350
	EES52754.1	putative fumarate reductase/succinate dehydrogenase	0.180	0.320	0.116			
	EES52755.1	succinyl-CoA synthetase, beta subunit	0.108	0.160	0.175			
	EES52756.1	succinyl-CoA synthetase, alpha subunit	0.024	0.078	0.087			
	EES52870.1	Pyruvate:ferredoxin oxidoreductase alpha subunit	0.324	0.370	0.407			
	EES52871.1	putative pyruvate:ferredoxin oxidoreductase beta subunit	0.156	0.192	0.495		0.0436	0.0423
	EES52872.1	Pyruvate:ferredoxin oxidoreductase gamma subunit	0.228	0.269	0.698		0.0097	0.0092
	EES52873.1	probable pyruvate:ferredoxin oxidoreductase epsilon subunit	0.120	0.160	0.582		0.0012	0.0010
	EES53316.1	probable pyruvate:ferredoxin oxidoreductase epsilon subunit	0.324	0.064	0.320	0.0000		0.0075
	EES53317.1	putative pyruvate:ferredoxin oxidoreductase gamma subunit	0.480	0.320	0.786			0.0088
	EES53318.1	Pyruvate:ferredoxin oxidoreductase beta subunit	0.348	0.452	0.786			
	EES53319.1	Pyruvate:ferredoxin oxidoreductase alpha subunit	0.432	0.680	0.931		0.0430	
	EES53330.1	phosphoenolpyruvate carboxylase	0.060	0.055	0.145			
	EES53418.1	Fumarate hydratase, class II	0.012	0.005	0.000			
	EES53484.1	Nitrogenase iron protein (NifH)	0.012	0.000	0.058			
	EES53485.1	Nitrogenase, molybdenum-iron protein alpha chain (NifD)	0.000	0.018	0.145		0.0468	
	EES53503.1	Nitrogen fixation protein (NifT)	0.000	0.000	0.029			
	EES53842.1	Isocitrate dehydrogenase (NAD(+))	0.204	0.087	1.658		0.0000	0.0000
	EES53843.1	probable isocitrate dehydrogenase (NADP)	0.120	0.041	1.833		0.0000	0.0000
	EES53955.1	malate dehydrogenase, NAD-dependent	0.084	0.059	0.000			
Iron oxidation	EES53608.1	cytochrome c, class I	0.036	0.018	0.000			
	EES53609.1	cytochrome c, class I	0.000	0.009	0.029			
	EES53762.1	cytochrome c assembly protein	0.024	0.018	0.029			
	EES54014.1	Cytochrome c oxidase, subunit I	0.480	2.288	0.262	0.0000		0.0000
	EES54017.1	probable cytochrome-c oxidase, subunit I	0.000	0.005	0.058			
	EES51436.1	Cytochrome 572	0.276	0.283	3.666		0.0000	0.0000
	EES51507.1	probable cytochrome c-554	0.000	0.046	0.000			
	EES51555.1	probable cytochrome C oxidase	0.024	0.100	0.029			
	EES51557.1	probable cytochrome-c oxidase	0.372	0.068	0.262	0.0000		
	EES51563.1	probable cytochrome C oxidase mono-heme subunit, partial	0.276	0.137	0.436			0.0165
	EES51589.1	Cytochrome-c peroxidase	0.108	0.137	0.029			
	EES51644.1	D-lactate dehydrogenase (Cytochrome)	0.012	0.032	0.029			
	EES51737.1	cytochrome bd ubiquinol oxidase, subunit I	0.000	0.087	0.000			
	EES51738.1	cytochrome d ubiquinol oxidase, subunit II	0.000	0.037	0.000			
	EES51901.1	cytochrome c, class I	0.000	0.018	0.000			
	EES52050.1	cytochrome c biogenesis protein	0.060	0.023	0.029			
	EES52053.1	cytochrome c assembly protein	0.156	0.137	0.495		0.0436	0.0036
	EES52139.1	cytochrome c, class I	0.000	0.037	0.029			
	EES52140.1	cytochrome c, class I	0.012	0.014	0.000			
	EES52416.1	Cytochrome b/b6, N-terminal domain	0.276	0.046	0.349	0.0001		0.0004
	EES52417.1	probable cytochrome b/b6, C-terminal	0.204	0.082	0.436			0.0005
	EES52419.1	probable cytochrome c, class I	0.120	0.064	0.233			
	EES52700.1	cytochrome B561	0.000	0.000	0.000	0.0000	0.0000	0.0000
	EES52848.1	Cytochrome b/b6, N-terminal domain	0.048	0.087	0.204			
	EES53128.1	Cytochrome-c oxidase, subunit I, partial	0.060	0.370	0.727	0.0001	0.0000	
	EES53555.1	cytochrome c, class I	0.060	0.945	0.000	0.0000		0.0000
	EES53578.1	probable Cytochrome c, NapC/NirT family	0.048	0.032	0.029			
Sulfur oxidation	EES51740.1	putative sulfide-quinone reductase	0.012	0.055	0.087			
	EES53159.1	putative sulfide-quinone reductase	0.012	0.009	0.058			
Proteasomes	EES53749.1	probable 20S proteasome alpha-subunit	0.024	0.183	0.000	0.0113		
	EES53750.1	putative 20S proteasome beta-subunit	0.072	0.164	0.320			
	EES53752.1	putative proteasome component	0.132	0.388	0.378	0.0078		
	EES52727.1	putative proteasome component	0.120	0.311	0.000			0.0044
	EES52729.1	putative 20S proteasome beta-subunit	0.060	0.192	0.000			
	EES52730.1	putative 20S proteasome alpha-subunit	0.024	0.132	0.058			
	EES52731.1	putative proteasome component	0.012	0.082	0.175			
Phage/virus stress	EES53469.1	CRISPR-associated protein, Cas4	0.000	0.005	0.029			
	EES53470.1	CRISPR-associated protein, Cas5	0.012	0.000	0.000			
	EES51830.1	CRISPR-associated protein, NE0113 family	0.036	0.005	0.000			
	EES53373.1	phage integrase family protein	0.048	0.000	0.000			
	EES53930.1	phage integrase family protein	0.036	0.009	0.262		0.0344	0.0001
	EES52912.1	phage integrase family protein	0.024	0.023	0.000			
	EES52639.1	phage integrase family protein	0.024	0.000	0.000			
	EES51420.1	phage integrase family protein	0.012	0.018	0.000			
	EES53680.1	phage integrase family protein	0.012	0.005	0.087			
	EES51665.1	phage integrase family protein	0.012	0.000	0.000			
	EES53290.1	phage integrase family protein	0.000	0.009	0.320		0.0001	0.0000
	EES52264.1	phage integrase family protein	0.000	0.005	0.029			
	EES51725.1	phage integrase family protein	0.000	0.000	0.087			

*Only those P value ≤ 0.05 are shown.
